# Social structure, opportunistic punishment and the evolution of honest signaling

**DOI:** 10.1371/journal.pone.0188249

**Published:** 2017-12-08

**Authors:** Robin Clark, Steven O. Kimbrough

**Affiliations:** 1 Department of Linguistics, University of Pennsylvania, Philadelphia, Pennsylvania, United States of America; 2 Department of Operations, Information, and Decisions, University of Pennsylvania, Philadelphia, Pennsylvania, United States of America; Universitat Jaume I, SPAIN

## Abstract

Honest signaling is generally taken to be a necessary pre-condition for a stable signaling system, because deceptive signaling at a high enough rate should cause receivers to ignore the signal, which in turn undermines the utility of sending signals. Deception is normally thought to occur because of benefits it has to the deceiver. This raises the question of why signaling systems should exist and persist over time, especially in cases in which the interests of the senders and receivers are not well aligned. Punishment has been seen as a way of imposing costs on deceptive signalers. We investigate the effects of opportunistic—that is, non-altruistic punishment—on the evolution of an honest signaling system. Our model is based on research done on social insects. We model a society of agents, divided into three castes differing in aggressiveness. Under severe punishment deception is indeed asymptotically eliminated. Under somewhat less severe punishment, deception persists and the rates of deception correlate with social structure. We find that social structure robustly mediates the level of deception under regimes of punishment and that this is evident except in the most stringent of punishment regimes.

## Introduction

A signaling system allows senders to alert receivers about properties of the world; a reliable signaling system is one in which the properties of the signal correlate well with properties of the world. In this paper, we will consider an extremely simple model of the evolution and maintenance of a reliable signaling system based solely on punishment of dishonest agents. Individual agents will have no memory for past behavior and no interest in their own truthfulness; instead, they are willing to punish agents who have signaled deceptively to them. We will see that agents must balance the strategic advantage of deception with the potential cost of being punished. In general, reliable signaling can be achieved if the punishment is sufficiently harsh. In systems with less stringent punishment, deception will persist and shows an interesting connection with social structure, as we will discuss below.

Our model is based on research done on social insects. [1, 2]. It is responsive to Gricean pragmatics [3], which requires conversational partners to obey maxims that require honest signaling. The Maxim of Quality requires a speaker to say what it believes to be true and not to say what it believes to be false; the Maxim of Quantity requires speakers to be as informative as required, but not more so. Grice’s maxims, then, predispose speakers to “tell the truth, the whole truth, and nothing but the truth.” In this paper, we will take the Maxim of Quality as a description of the outcome of a dynamic process. We will show, using a Agent-Based Model, (i) that honest signaling emerges when agents opportunistically punish deception and (ii) that deception is part of a mixed-strategy equilibrium. In particular, in a highly unequal society, social structure promotes the persistence of deceptive signaling, even under relatively stringent punishment.

Reliability—or “honesty”—is often taken to be essential to any communication system (see [4–6]; among many others). The absence of reliability would mean that the signals do not correlate well with properties of the world; in that case, a receiver would be well advised to ignore the signal since it carries little information about the actual world. A signaler, then, would have little interest in wasting the resources to send a signal that will, in any event, be ignored. Human communication also requires some level of reliability in order to be useful; the need for honesty has been codified by the “Maxim of Quality” [3] which requires speakers to be truthful. The evolution of honest signaling is important for understanding the evolution of both animal and human communication. We return to this in the “Discussion” section.

If signalers are sending signals for their own strategic benefit, that is they seek to manipulate the receiver’s behavior [7, 8], then the maintenance of reliability is orthogonal to the main goal of persuasion and influence. Signalers are free to use deception in order to achieve their goals and, naturally, receivers are free to ignore signals they suspect are deceptive. Such systems, which include natural language, employ “cheap talk” [9]; by the above argument, we would expect cheap talk systems to be unstable, such instability being witnessed by cases of actual language change [10] [11]. Nevertheless, it is the case that natural language, despite the deception, is taken to be largely reliable. A system that is too unreliable will be ignored, so it is in the sender’s interests to maintain some level of honest signaling—enough to maintain some level of trust in the system—while occasionally using deception to exploit this trust.

The overall reliability of natural signaling systems, particularly ones which, like natural language, verge on cheap talk, is worthy of investigation [12]. We might, for example, hypothesize that costly signaling could be a guarantor of reliability. For example, the *Zahavi handicap principle* [13] suggests that honest signaling will tend to come at a price to the signaler. This price could be accrued as *efficacy costs*, which are costs associated with the physical production and transmission of the signal, or *strategic costs*, which involve costs that are external to the production of the signal [4]. Peacock fans, to take a famous example, are costly to produce and are taken as a prototypical example of a handicap: the elaborate fan truthfully signals that its bearer is fit. Equally, as [4] suggests, honesty could be assured by *strategic costs*; the signal itself is not costly to produce, but the consequences of an unreliable signal could have a negative impact on the signaler. It may be, for example, that a deceptive signaler, once caught out, might suffer some punishment for his misbehavior [14]. This work is, in part, intended as a contribution to the study of the role that deception plays in communication [15–21]

Studies of signaling in the paper wasp, *Polistes dominula*, have suggested that social punishment might be an effective means of ensuring reliable signaling [1, 2]. (We thank an anonymous reviewer for the following clarifying comment: “signs in the *clypeus* to signal aggression level are apparently only a female characteristic. Males signal their aggressiveness with abdominal spots.”) The wasps establish relative social dominance via aggressive encounters that include mounting. As it happens, these wasps have acute vision and are capable of making fine visual distinctions, so that they are able to recruit markings on their clypeus to signal aggression level: More aggressive wasps have “broken” facial patterns, while less aggressive wasps have a single mark or no mark at all. Such a system, given that it is reliable, would allow the wasps to quickly assess the agonistic abilities of a potential rival. Our work was inspired by [1, 2] but is not intended to be a model of wasp behavior. Instead, we intend to model a set of incentives and their effects on stylized agents, which we will occasionally refer to as “wasps” to recognize the inspiration of the model. For the most part, though, we will refer to these entities as “agents.”

The marking system can also be used to impose deception on the wasps by altering the patterns on the clypeus [1]. Thus, a less aggressive wasp can be made to bluff by appearing more aggressive by giving it a broken clypeus badge; such deception might be to the wasps advantage, supposing that it could scare away at least some potential competitors. Equally, a more aggressive wasp can be made to appear less aggressive by joining its broken badge into an unbroken one; again, some advantage might be had in doing so since it could seduce some competitors to drop their guard. An aggressive exchange between wasps will, of course, reveal the actual aggression level of the players and, as a result, out any deception [2]. The marking system employed by paper wasps lacks any obvious efficacy costs.

These studies intriguingly occasion a more general question. Is is well known that honesty promoted by punishment of dishonesty can under some circumstances extinguish dishonesty. Are there mitigating conditions under which, even with strong levels of punishment for deception, deception can be preserved in the face of honest signaling? Our aim is not to model any particular biological society. Instead, inspired by the wasps studies, we wanted to investigate the population dynamics of honest signaling when promoted by punishment. We conjecture that social stratification can be an important mitigating factor in these dynamics. In this paper we study these dynamics by developing a simple agent-based model of a society in which honest and dishonest signaling, punishment for dishonesty, and social structure are present.

We might imagine that both types of wasp have some basis for employing deception, which would inevitably reduce the reliability of the signaling system. What force maintains the quality of the signaling? [1] hypothesized that social punishment would reduce cheating. Deceptive wasps would be punished after aggressive encounters. [2] showed that a mismatch between signal and behavior can reveal deception and be used as a basis for punishment. A unique aspect of the model developed here is that agents are indifferent to their own truthfulness but are willing to punish deception in others; a case can be made that social punishment provides a way of imposing strategic costs on a signaling system, beyond moral considerations and empathy [16]. It remains to be seen, however, what the population dynamics of social punishment are. It is possible that, given the incentives to deceive in some cases, deceptive signaling would persist in a population even in the presence of strong social punishment. Our model is a stylized society; it is not our intention to model any particular naturally occurring society. Doing that with any fidelity in an Agent-Based Model is well beyond the scope of the available data and, indeed, technology. Instead, our model seeks to explore the consequences of certain incentives and behaviors, which are plausibly present in both wasp and other societies. No doubt, other factors matter and may well overwhelm the factors we are exploring. Nonetheless, we believe that this basic model is informative with regard to the incentives and disincentives for deception. It is well known that punishment will tend to favor honestly, but what is new here is the exploration of the interactions between different levels of punishment operating among distinct social groups (“castes”). As described in the paper, the results are complex and surprising.

We investigate this question using an Agent-Based Model, described in the “Materials and methods” section; this model will allow us to study the consequences of punishment in an evolutionary model of behavior; we look, in particular, at “opportunistic” punishment by which we mean punishment that imposes no cost on, but rather a tangible reward to, the agent who punishes the deceiver. Our results, in the “Results” section, indicate that deception can be quite persistent even under harsh punishment. What is more, deception is conditioned by the social structure, with each class displaying a characteristic pattern of evolution in the signaling system. We conclude, in the “Discussion” section, with an examination of the connection between honest signaling and social structure, as well as the role of social structure in explaining the social order.

## Materials and methods

In order to test the effectiveness of social punishment in the evolution of reliable signaling, we developed an Agent-Based Model in which individuals played a dominance game: Based on the (possibly deceptive) signal of a potential opponent, an agent could attempt to dominate another individual, as described below. Once the outcome of the contest was determined, the winner could extract resources from the loser; if the loser of the contest had used a deceptive signal, the winner could extract additional resources from the loser.

Underlying the model is the assumption that agents are indifferent about the reliability of their own signals. If deceptive signals result in higher payoffs, then we would expect agents to prefer deception. Agents are, however, willing to punish signalers who attempt to deceive them; in other words, although agents are indifferent to their own deception, they are sensitive to deception directed at them by others. This is consonant with the idea that the agents are self-interested.

In addition, following [1], we recognize that there are different types of deception:

1. **Bluffing:** A weak agent signals that it is stronger than it actually is, presumably to frighten away potential rivals and by doing so maintain its resource level;
2. **Seduction:** A strong agent signals that it is weaker than it actually is, presumably to draw weaker agents into dominance games that they can’t win and extract their resources.

In the original [1] study, the wasps came in two sorts: Highly aggressive wasps, associated with a signal (clypeus markings) which indicated their aggression level and less aggressive wasps, also associated with a signal that indicated their aggression level. As a result, high aggression wasps can only deceive by seduction and low aggression wasps can only deceive by bluffing. By the very structure of the system, individuals cannot choose the nature of their deception.

Our primary question is whether or not punishment can drive deception out of a signaling system; ancillary to this are questions concerning the type of deception available to the agents. If there are two types of agents, strong and weak, then seduction will be available solely to the strong agents and bluffing will be available solely to the weak agents; once their roles are set, the agents have no possibility to select the type of deception they might employ. The different types of deception in the system, however, might have different evolutionary dynamics with respect to punishment. In order to explore this question, we developed a society with three types of agents instead of two:

1. High aggression: These are the strongest type of agent, with the greatest resources. Their honest signal is “red” although they can seduce in two ways: By signaling that they are medium aggression agents or by signaling that they are low aggression agents.
2. Medium aggression: These are less aggressive than the “red” agents with moderate resources. Their honest signal is “yellow” although these agents may bluff by signaling that they are high aggression agents or seduce by signaling that they are low aggression agents.
3. Low aggression: These agents have the lowest level of aggression and the least amount of resources. Their honest signal is “green.” They may bluff in two ways: By signaling that they are medium aggression agents or by signaling that they are high aggression agents.

For present purposes, we will refer to the different types of agents in our model as “castes.”

We begin with a population of 3,000 agents partitioned more or less equally into the three castes. Agents in the high aggression caste are endowed with 100 resource points; agents in the medium aggression caste are allotted 75 resource points; agents in the low aggression caste are given 50 resource points. When an agent’s resources sink to less than 10% of its original endowment, it is replaced by a new agent of the same caste, with a new initial endowment; the signaling strategy (honest or dishonest; if dishonest, the type of deception employed) will be determined by a tournament, as described below. In the initial simulation, 85% of the agents are deceptive signalers; the question is whether punishment can significantly reduce or eliminate deception. (We note that our purpose was to investigate the degree to which truth telling can completely eliminate deception under punishment in a socially stratified society. The key issue from our evolutionary dynamics point of view is whether small levels of truth telling can expand in the population at the expense of liars. Thus, we are primarily interested in exploring the dynamics incident upon initially high levels of deception. We briefly explored, of course, values other than 85%, but did not see interesting changes in the dynamics and so we do not report these.)

The agents are paired off at random in a game of incomplete information [9, 22]; that is, the agents are unsure of the payoff structure of the strategic interaction in which they are engaged because the signal their opponent sends them correlates poorly with their actual type; they do not have reliable information about their opponent’s potential moves. The game itself can be divided into four stages:

1. Signaling
2. Fight or flight
3. Payoffs and punishment
4. Replication

When the agents are paired, one agent—the focal agent—receives a signal from its opponent. The focal agent then decides whether or not to engage its opponent in a contest; if it decides not to engage its opponent, it flees. This can happen when the opponent signals that it is more aggressive than the focal agent so that there is a tendency for relatively low aggression agents to flee in the face of high aggression agents. It can also happen when two agents have the same level of aggression, but the resources of the focal agent are relatively low. The ratio of the focal agent’s current resource level to its initial endowment is computed; the lower the ratio, the likelier the focal agent is to flee. Fleeing requires the focal agent to pay a metabolic cost that is deducted from its resources. Although the cost incurred by the focal agent is relatively small, fleeing is not a viable strategy in the long run, since agents must maintain their resources above a certain level in order to survive.

The next stage, which occurs when the focal agent elects to engage its opponent, is the actual battle between the agents. The rules are quite simple; if the agents have different levels of aggression, the one with the higher aggression level wins: High aggression agents defeat medium and low aggression agents; medium aggression agents defeat low aggression agents. If the two agents have the same level of aggression, then the agent with the greater level of resources wins; if they have the same resources, the winner is selected at random.

Once the fight phase is completed and an actual winner of the encounter is determined, it is time to distribute payoffs and mete out punishments.

1. Low aggression agents take 25% of their honest opponent’s resources and 50% of their dishonest opponent’s resources;
2. Medium aggression agents take 50% of their honest opponents resources and 75% of their dishonest opponent’s resources;
3. High aggression agents take 75% of their honest opponent’s resources and 100% of their dishonest opponent’s resources.

Notice that, in all cases, more resources are extracted if the losing agent is a deceptive signaler; naturally, the winning agent receives more resources from a deceptive signaler than it would from an honest signaler. Dishonest signalers are, then, punished when they lose a battle, even if the winner is itself a dishonest agent.

When an agent’s resources drop below 10% of its original resources, it is no longer viable and is replaced by a new agent from the same “caste” (aggression level). The replication relies on a simple tournament: Two agents are selected at random from the defunct agent’s aggression level. The agent with the higher level of resources wins the tournament and its signaling strategy is copied by the new agent. This tournament structure is particularly unforgiving for weak strategies since, as a strategy becomes less frequent, it becomes less and less likely that it will be picked by the tournament.

The above framework is particularly stringent; if deceptive signaling is harshly punished and, as a result, becomes rare in the population, it should be driven out, resulting in a population of honest signalers. Our question, then, is whether deceptive signaling will be eliminated from the population due solely to prosocial punishment.

We conducted a full-factorial sweep at four levels of the four main parameters in the model, amounting to 4^4^ distinct parameter settings, each with 30 replications and run for 2,000,000 ticks. In what follows we report on a representative subset. To the best of our knowledge overall the behavior did not differ dramatically from the parameter settings we discuss here. The reader, of course, may use our code to conduct new runs. The four main parameters are:

1. aggressionResources.
  Each agent is given a level of resources upon creation and a metabolism. The resources given depend upon the agent’s level of aggression. In the default setup, low aggression (caste 0) agents have 50 units of aggressionResources, medium aggression (caste 1) agents have 75, and high aggression (caste 2) agents enjoy 100.
2. metCostFactors.
  This parameter represents the metabolic rate, or cost, for each agent. In the default setup, low aggression (caste 0) agents have a rate of 0.05 units, medium aggression (caste 1) agents have a rate of 0.075, and high aggression (caste 2) agents have a rate of 0.1. At each tick of the clock a focal agent (“the player”) is chosen at random as is another agent (“the counter player”). They encounter each other but only the focal agent’s resources are affect. If the agent flees combat, the agent’s metabolism uses of this portion of its aggressionResources. Agents whose level of resources fall enough die of starvation. More aggressive agents have higher metabolism rates. We emphasize that metCostFactors are not modeling agent metabolism. metCostFactors exist in the model in order to force agents to fight, at least sometimes, when challenged. Thus we eliminate the strategy of agents simply running away when challenged, as this would make the system uninteresting.
3. resourceHonestAppropriations.
  This is the proportion of resources taken from defeated honest agents. In the default setup, low aggression (caste 0) agents take 25% of the defeated agent’s resources, medium aggression (caste 1) agents take 50%, and high aggression (caste 2) agents 75%.
4. resourceDisHonestAppropriations.
  This is the proportion of resources taken from defeated dishonest agents.
  In the default setup, low aggression (caste 0) agents take 50% of the defeated agent’s resources, medium aggression (caste 1) agents take 75%, and high aggression (caste 1) agents 100%.
  Thus, comparing resourceHonestAppropriations and resourceDisHonest-Appropriations, under the default settings dishonesty is severely penalized compared to honesty among the defeated.

Two randomly drawn agents, theParty and theCounterparty, confront each other asymmetrically, focused on theParty agent. If theParty’s resources are high enough or if it receives a peaceable signal from theCounterparty, then theParty fights; otherwise it chooses to flee.

If theParty chooses to flee, it pays metabolic costs according to metCostFactors. If theParty chooses to fight an outcome of victory or defeat is determined (in battleOutcome()). truthOrConsequences() records the consequences. Only theParty’s resources are affected. They are adjusted up or down according to resourceHonestAppropriations and resourceDisHonestAppropriations.

## Results

**Fig 1:**
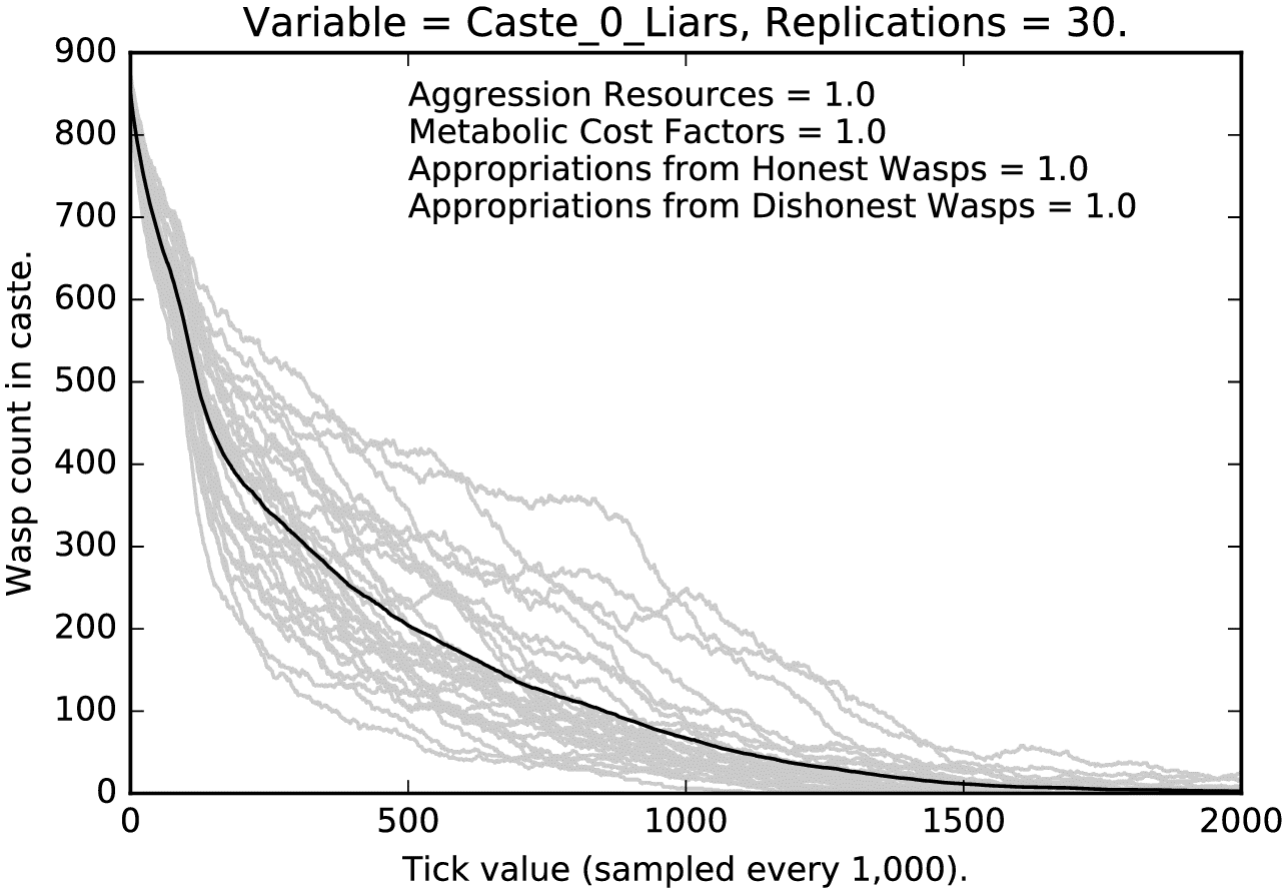
Default parameter settings: Counts of caste 0 liars over time, with 30 replications (in grey). (Mean counts in black.)

**Fig 2:**
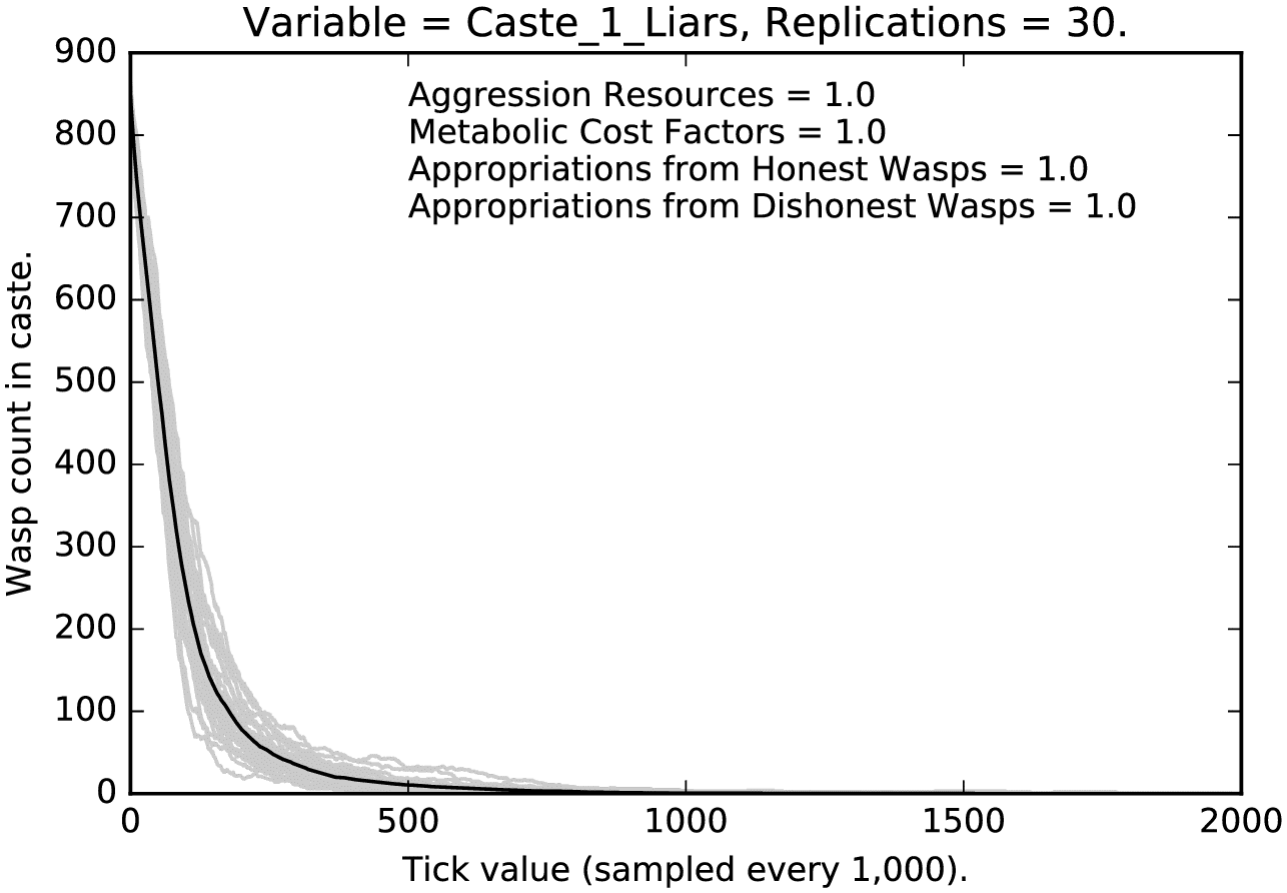
Default parameter settings: Counts of caste 1 liars over time, with 30 replications (in grey). (Mean counts in black.)

**Fig 3:**
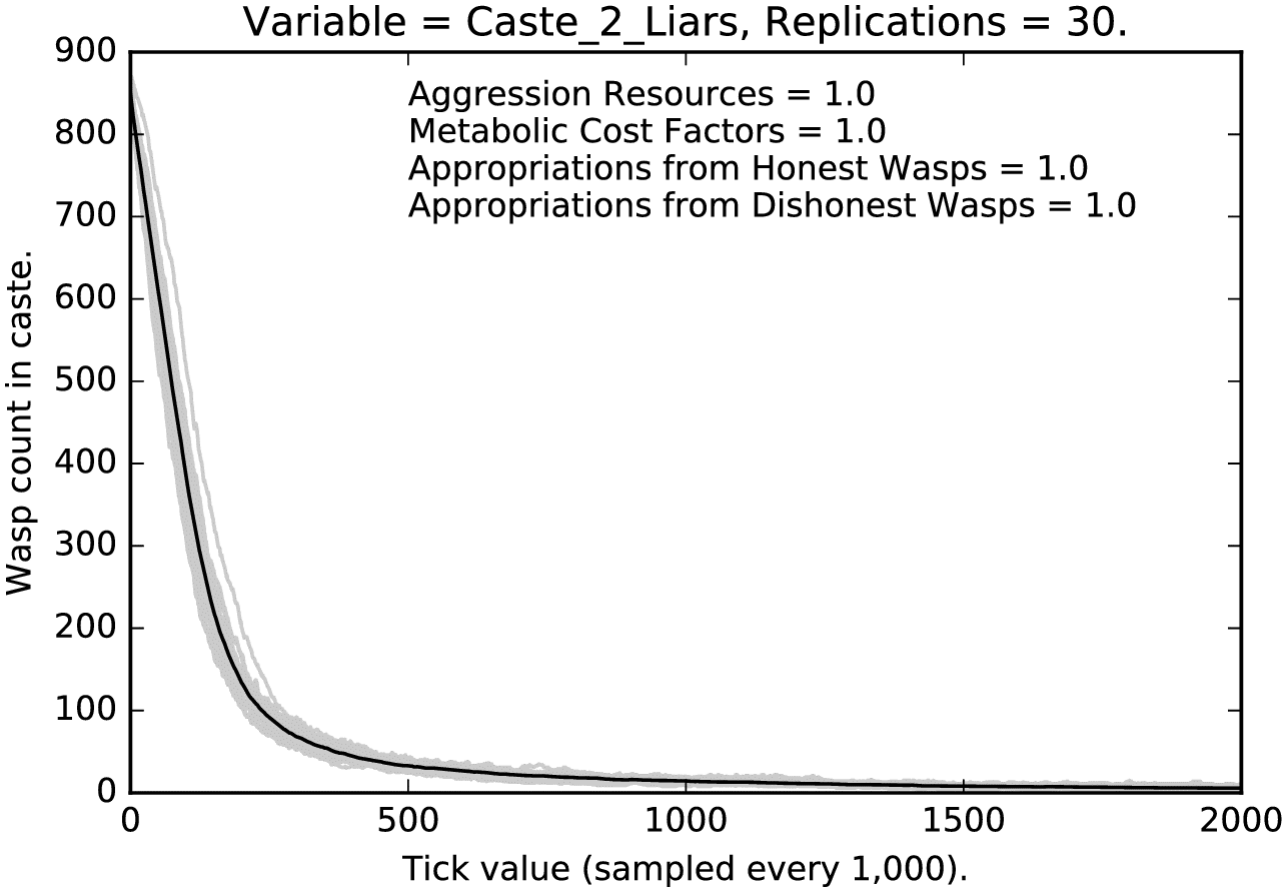
Default parameter settings: Counts of caste 2 liars over time, with 30 replications (in grey). (Mean counts in black.)

Figs 1, 2 and 3, using the default values for the four parameters (above) tell a basic story, viz., that (i) with very strong disincentive for deception, it weakens dramatically, and (ii) the degree of weakening varies with caste, so social structure matters. Notice as well, the variance across runs is comparatively high for caste 0, is tight for caste 1, and very tight for caste 2. In each of 30 replications we let the simulation run for 2,000,000 ticks so in each run we expect each agent along with its descendants to be picked as theParty about 666 times. (These points apply to all of the runs discussed in this paper.) In the present case, where punishment is at its highest, liars are almost entirely eliminated on average for each caste. However, the castes approach this state at distinctly different rates. Caste 1 is the fastest, followed closely by caste 2, then caste 0. Indeed, as Fig 1 indicates, while even after 2,000,000 ticks in some runs a noticeable number of liars remain in caste 0, it is clear that deception is being purged from the population, albeit rather slowly. Note that dishonest signaling is eliminated more quickly in castes 1 and 2.

Over the 2,000,000 ticks and on average across the 30 replications, caste 0 experienced 12,700 deaths (and rebirths). The average age at death was 148,663.7 ticks. For caste 1 the numbers are 9,957 deaths and average lifespan of 122,096.8 ticks, and for caste 2 they are 10,226 and 144,480.4 ticks. Thus, in terms of longevity, the caste 0 agents live the longest, then the caste 2, and then the caste 1.

**Fig 4:**
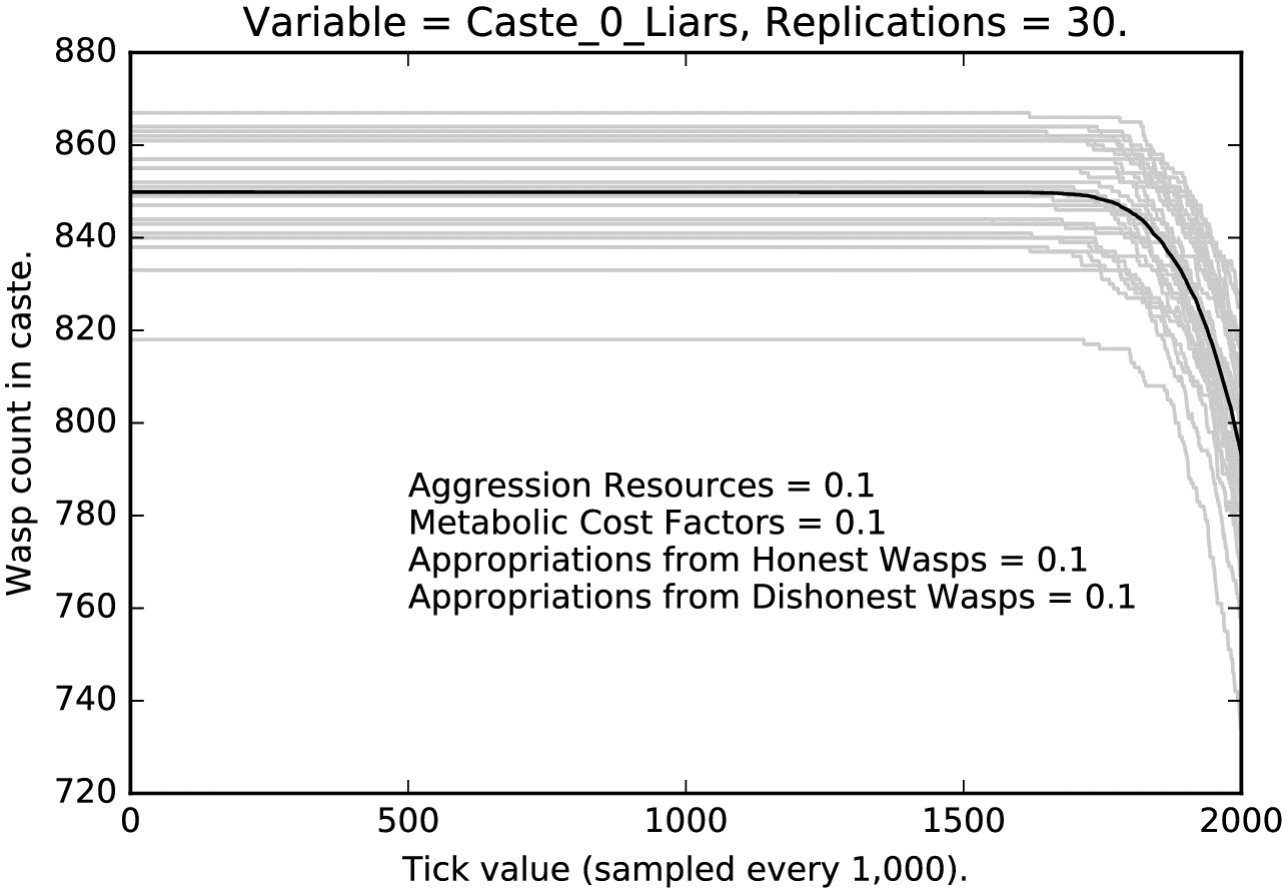
Minimal punishment parameter settings ([0.1, 0.1, 0.1, 0.1]): Counts of caste 0 liars over time, with 30 replications (in grey). (Mean counts in black.)

**Fig 5:**
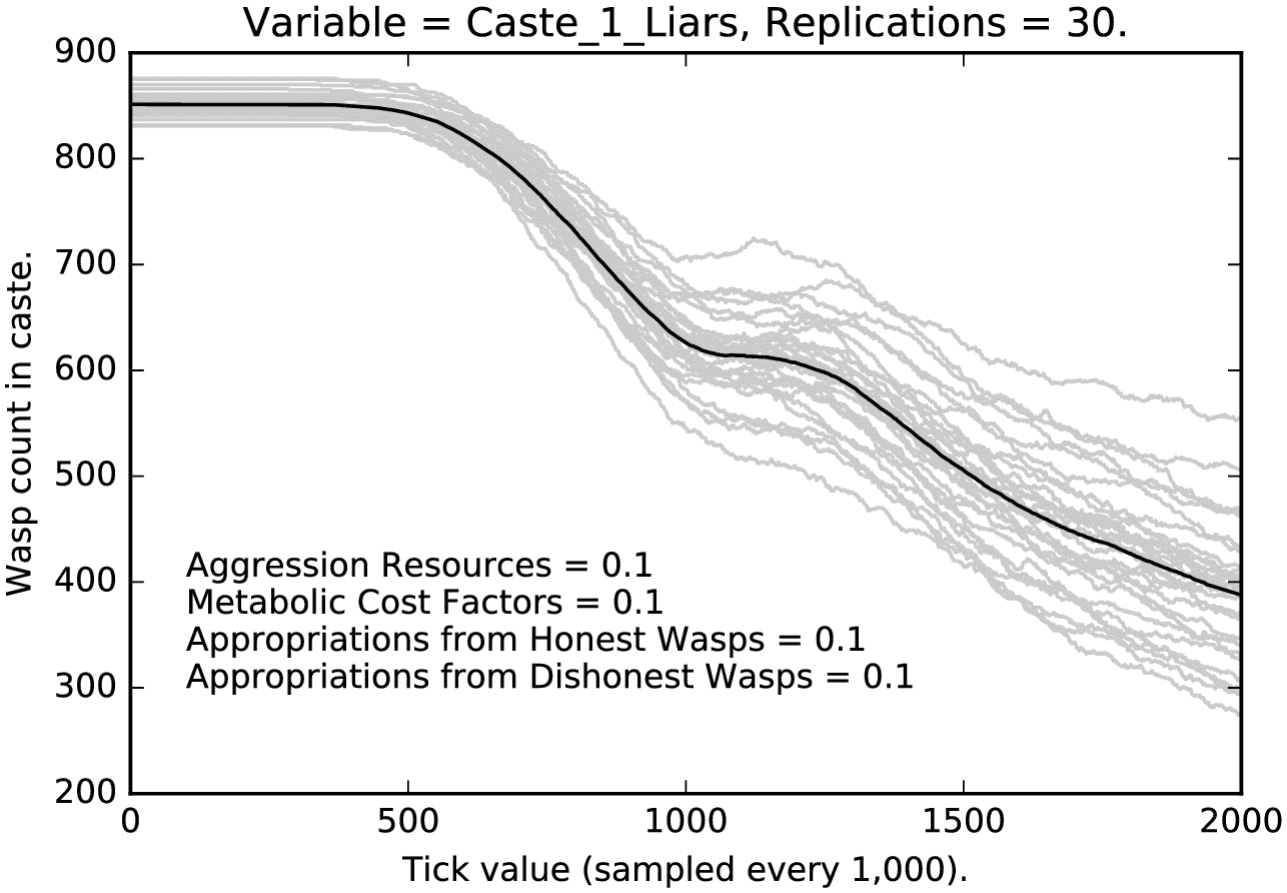
Minimal punishment parameter settings ([0.1, 0.1, 0.1, 0.1]): Counts of caste 1 liars over time, with 30 replications (in grey). (Mean counts in black.)

**Fig 6:**
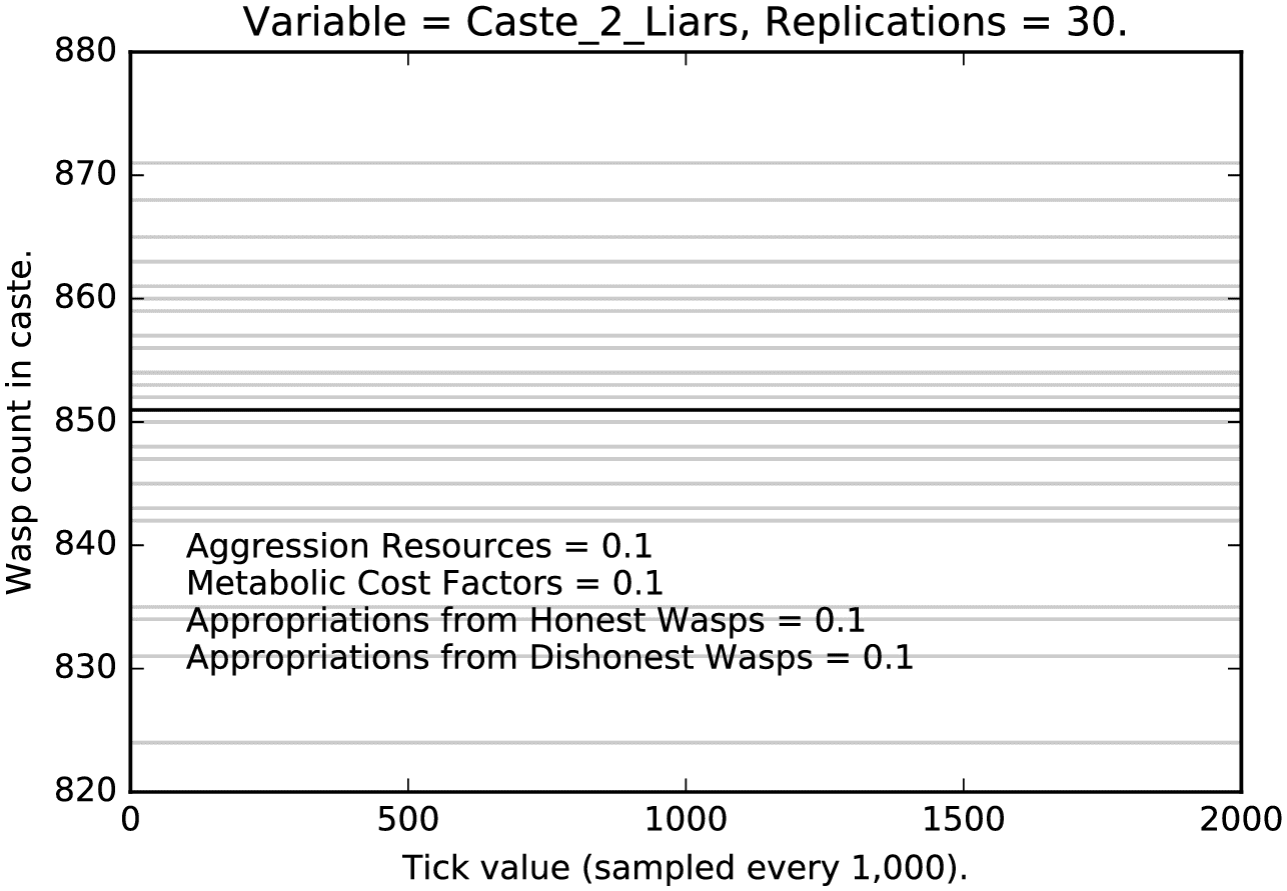
Minimal punishment parameter settings ([0.1, 0.1, 0.1, 0.1]): Counts of caste 2 liars over time, with 30 replications (in grey). (Mean counts in black.)

Figs 4, 5 and 6 present the corresponding results from the parameter setting in which each of the four parameters being explored have been set to 0.1 × their default values (indicated by 1.0) and so represent behavior with very light punishment. The behavior is now rather different, although recognizable. Again, caste 1 agents have the strongest decline in deceptive signaling. Caste 0 is stable with about 85% deception until about 1,800,000 ticks at which point it declines rapidly, but modestly. Caste 2 shows no change at all. Once a liar, always a liar (at least within the time horizons of our runs).

Over the 2,000,000 ticks and on average across the 30 replications, caste 0 experienced 235 deaths (and rebirths). The average age at death was 1.904540e+06 ticks. For caste 1 the numbers are 2235 deaths and average lifespan of 724,298.9 ticks, and for caste 2 they are 1 death 1646902 ticks. Thus, in terms of longevity, the caste 0 agents who died during the runs live on average the longest, then the caste 2, and then the caste 3.

By way of interpreting these results, which appear at the two extremes of the parameter space, we make the following observations:

1. We see a clear effect due to the relative severity of the social punishment imposed:
  - Under the most severe punishment regimen, lying is nearly eliminated, although some lying persists even after two million ticks, in the lowest and the highest castes (Figs 1 and 3); note that lying is entirely eliminated from the middle caste (Fig 2);
  - Under the least severe punishment regimen, lying clearly persists in all the castes; the variance of the rate of lying is quite high; note that the lowest caste shows little change in rate until about 175 million ticks (see Fig 4), the highest caste shows no change in rate (for reasons we will discuss below; see Fig 6), while the middle caste still shows signs of selection against deception (see Fig 5).
2. In each of the three different castes it can take a significant amount of time to converge to an apparent equilibrium (although the middle tends to converge quite quickly in most cases); note that for the highest caste in the most lenient punishment regimen, there is little evidence of change in the rate of deceptive signaling.
3. The three different castes show characteristically different paths over the course of the simulation; the highest caste preserves the highest rate of deception even under stringent punishment (see Figs 3 and 6), the lowest caste shows persistence of deception, although the rate deception decline is greater than that of the highest caste (see Figs 1 and 4); the middle caste purges liars at the highest rate, converging to completely honest signaling (see Figs 2 and 5).
4. The above points refer to the mean rates of deception. If we look at the signals actually being sent, we get a corroborating pattern (see the Discussion of intermediate punishment below).
5. If we consider the variance of the rates of usage of particular signals, we again see a repeated pattern that reflects social structure.

As noted above, we have systematically sampled a large number of parameter values from the [1.0, 1.0, 1.0, 1.0] …[0.1, 0.1, 0.1, 0.1] parameter space. Results are quite robust throughout. We now report in detail results for the [0.5, 0.5, 0.5, 0.5], which are quite representative.

### Intermediate punishment

**Fig 7:**
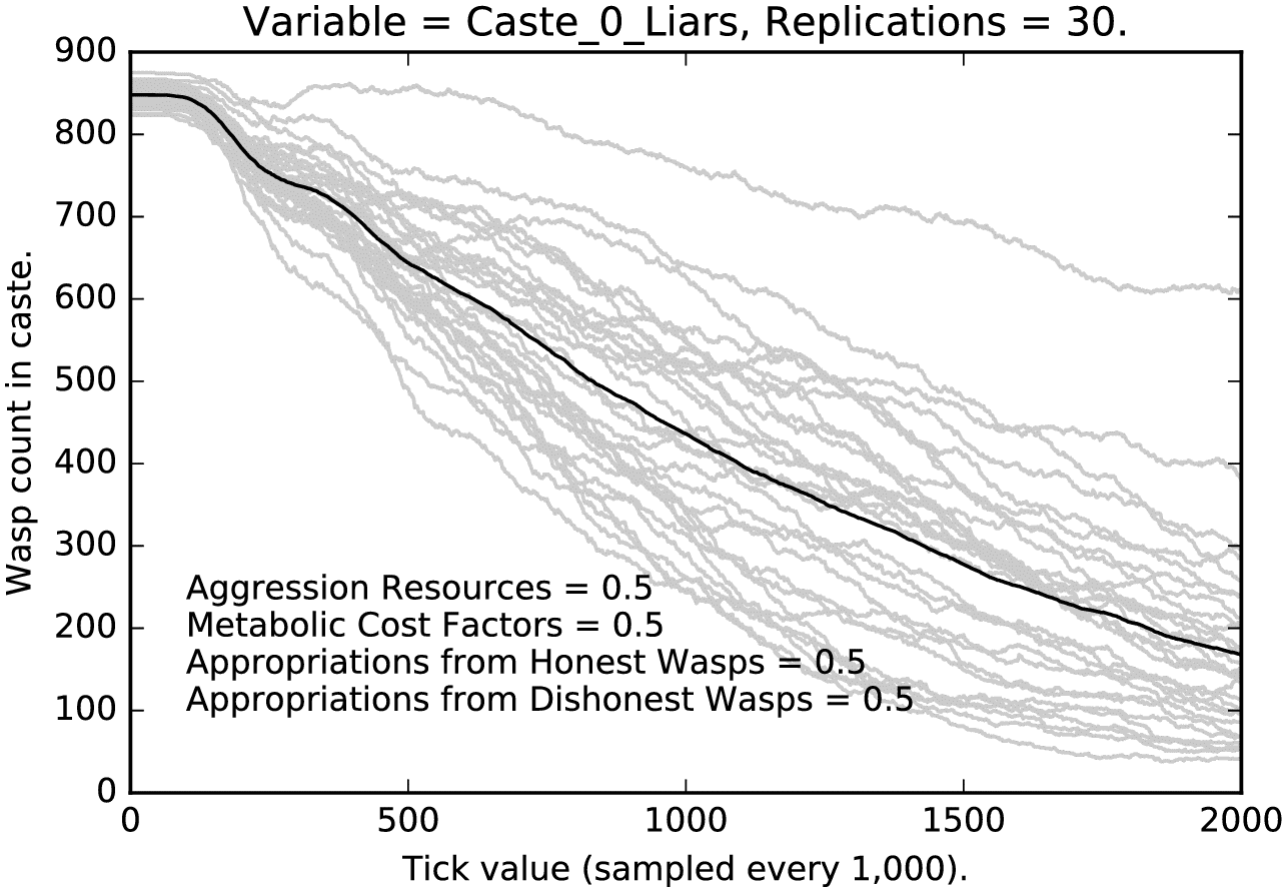
Intermediate punishment parameter settings ([0.5, 0.5, 0.5, 0.5]): Counts of caste 0 liars over time, with 30 replications (in grey). (Mean counts in black.)

In this subsection, we will compare the behavior of the most stringent regimen, with a regimen where punishment, etc., have been reduced by half. First, compare the relative stringent selection observed in Fig 1 for the lowest caste of agents with the less stringent regimen shown in Fig 7. The two cases resemble each other qualitatively, but with much stronger selection for honest signaling given the stringent punishment in the earlier case, as opposed to the weaker selection shown in Fig 7. Notice that many more deceptive signalers remain after 2 million interactions and that the variance of the deceptive signaling grows over time.

**Fig 8:**
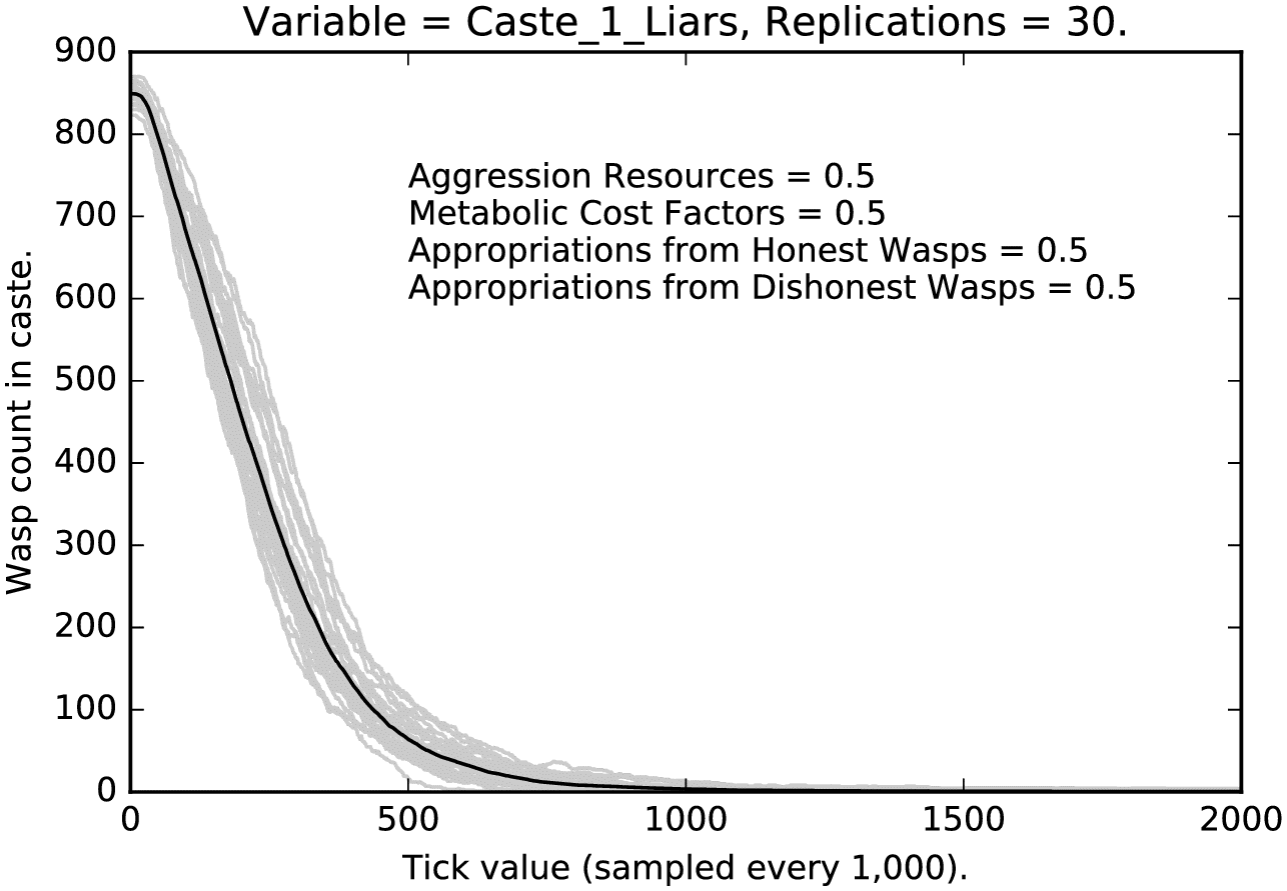
Intermediate punishment parameter settings ([0.5, 0.5, 0.5, 0.5]): Counts of caste 1 liars over time, with 30 replications (in grey). (Mean counts in black.)

Compare Fig 8 with Fig 2. In the former case, under the most stringent regimen, selection for honest signaling is quite strong; in Fig 8, selection is not as strong, but it is still the case that deceptive signaling is extinguished for the middle caste. This is presumably because of the difference in social positioning between the low caste agents and the middle caste agents.

**Fig 9:**
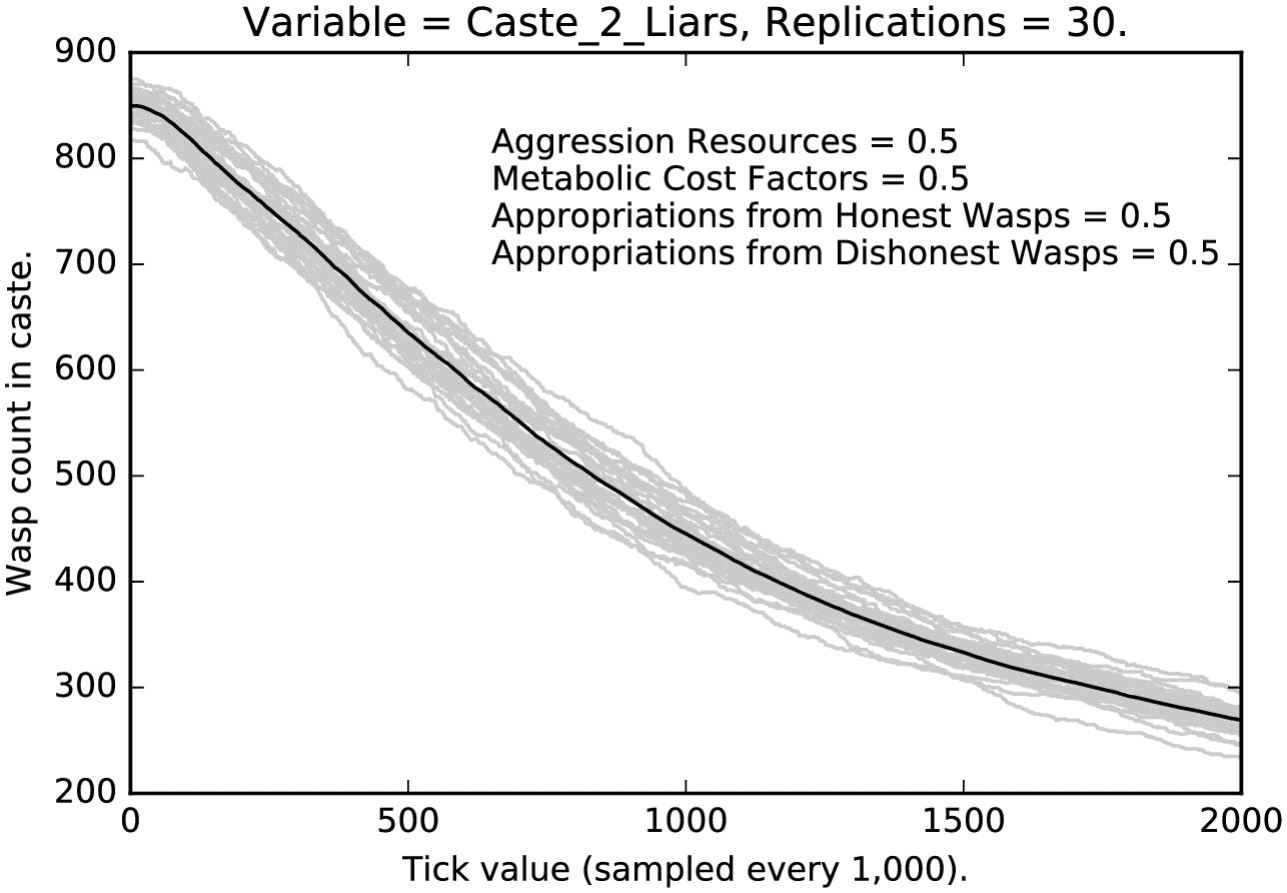
Intermediate punishment parameter settings ([0.5, 0.5, 0.5, 0.5]): Counts of caste 2 liars over time, with 30 replications (in grey). (Mean counts in black.)

Next compare the results for the highest caste of agents. Fig 9 shows a decline in the level of deceptive signaling, although about one third of the agents are still deceptive signalers under the milder punishment schedule; compare this with the stringent regimen shown in Fig 3, where deception is nearly extinguished. Notice, also, that the variance of deceptive signaling is lower for high caste agents as compared to the low caste agents in Fig 7, but it is higher than the variance in signaling displayed by the middle caste in Fig 8. In other words, each caste has its own unique signature.

**Fig 10:**
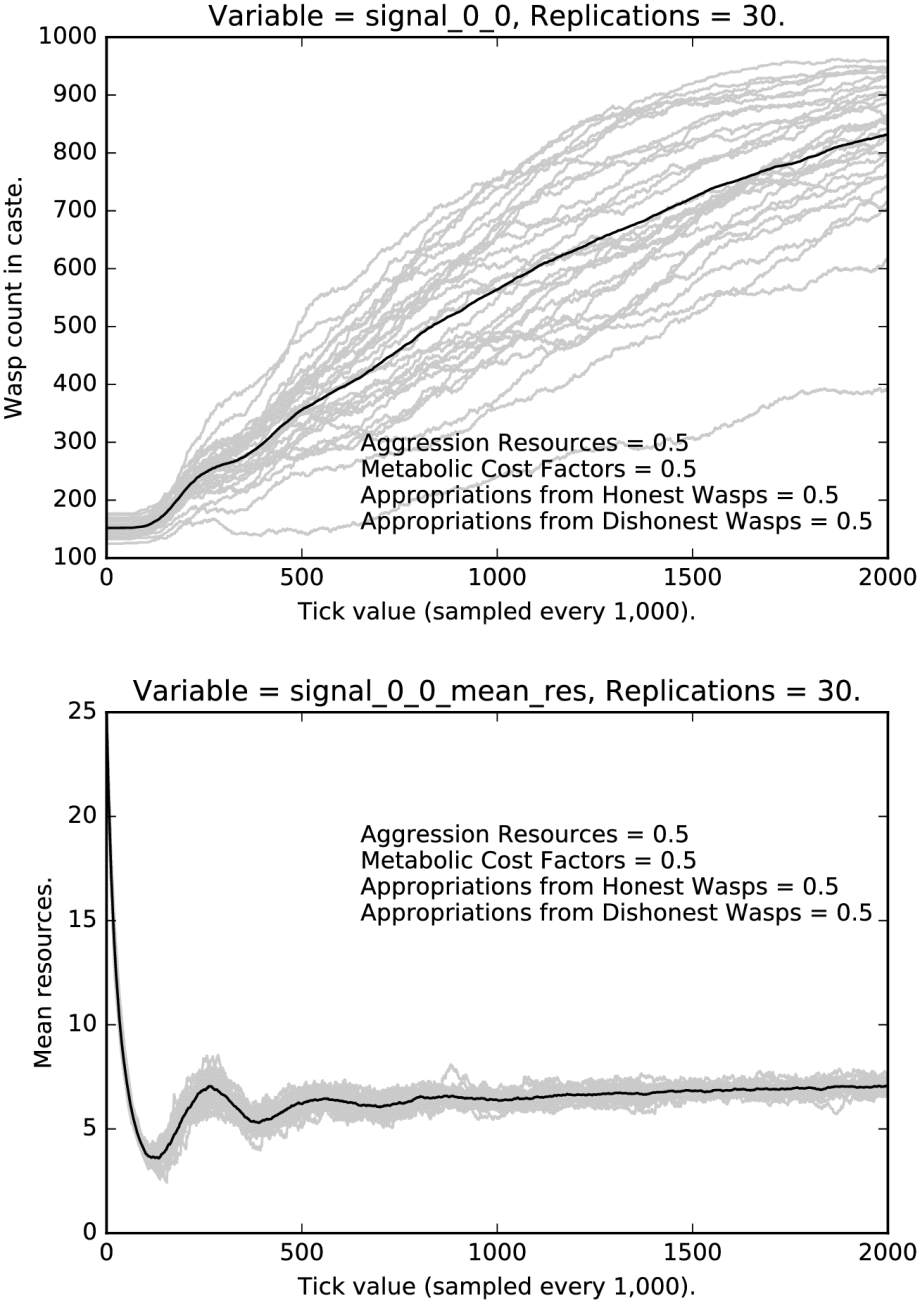
Top: Intermediate punishment parameter settings ([0.5, 0.5, 0.5, 0.5]): Counts of caste 0 0 signalers over time, with 30 replications (in grey). (Mean counts in black.) Bottom: Intermediate punishment parameter settings ([0.5, 0.5, 0.5, 0.5]): Mean resources of caste 0 0 signalers over time, with 30 replications (in grey). (Mean counts in black.)

**Fig 11:**
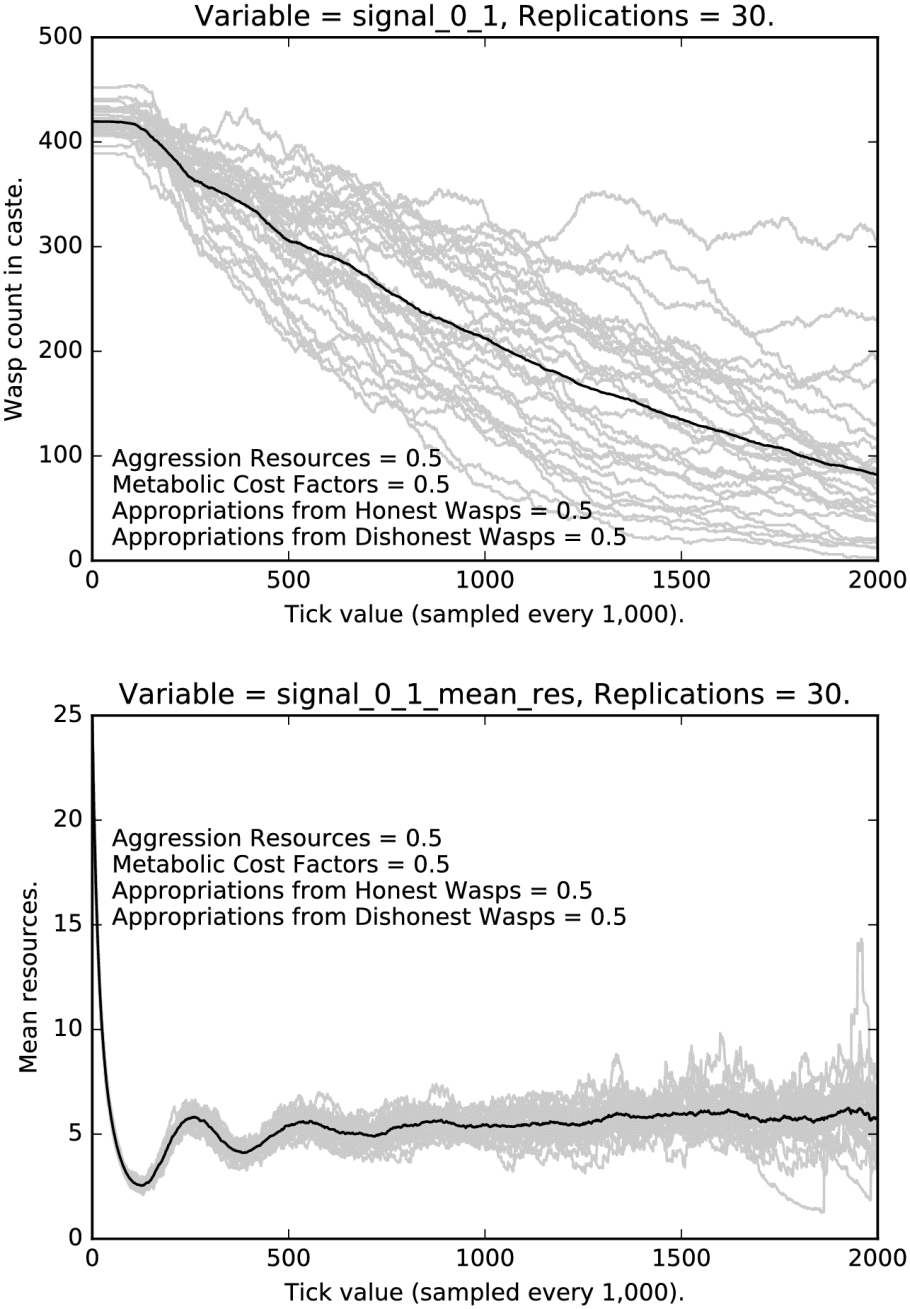
Top: Intermediate punishment parameter settings ([0.5, 0.5, 0.5, 0.5]): Counts of caste 0 1 signalers over time, with 30 replications (in grey). (Mean counts in black.) Bottom: Intermediate punishment parameter settings ([0.5, 0.5, 0.5, 0.5]): Mean resources of caste 0 1 signalers over time, with 30 replications (in grey). (Mean counts in black.)

**Fig 12:**
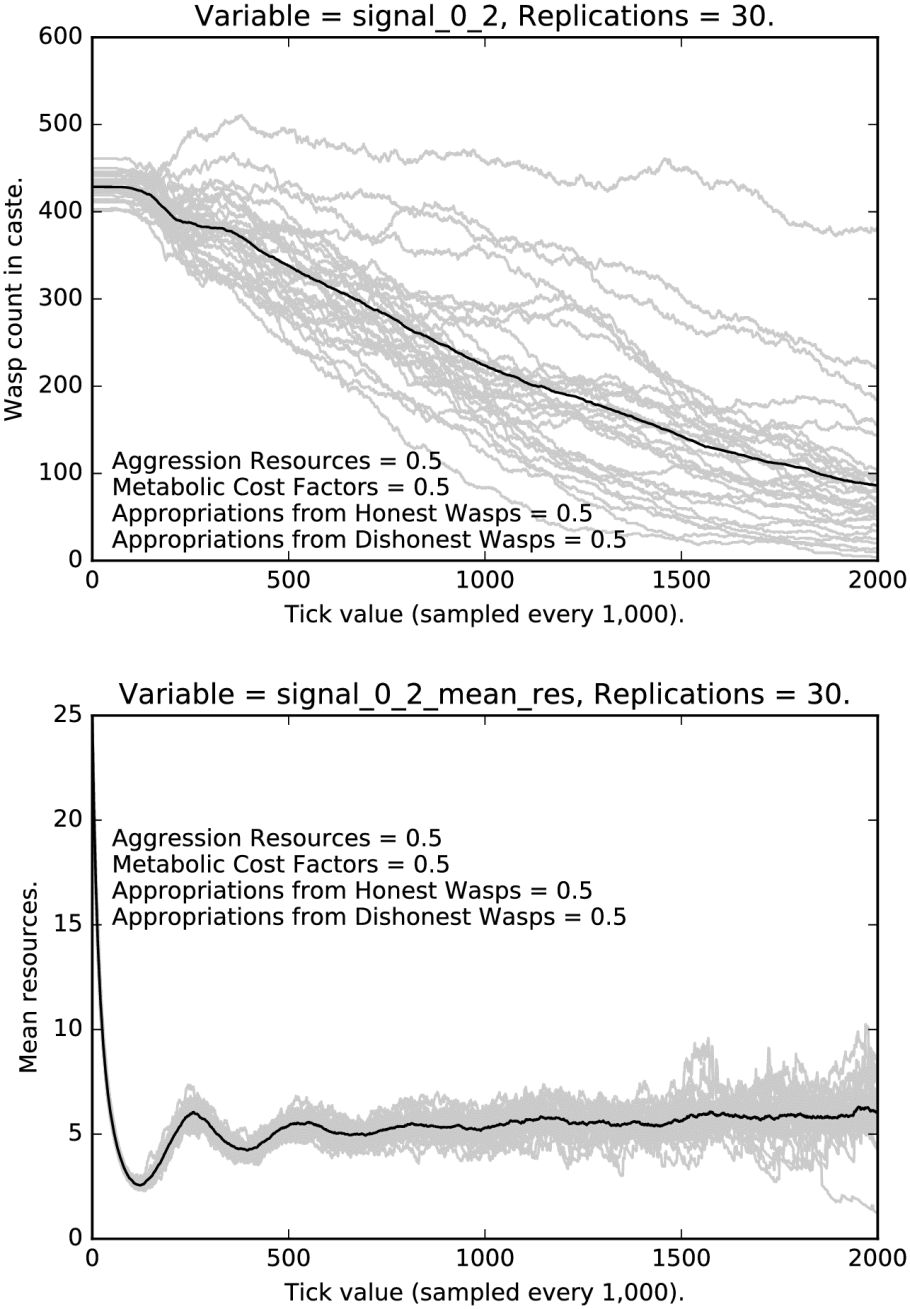
Top: Intermediate punishment parameter settings ([0.5, 0.5, 0.5, 0.5]): Counts of caste 0 2 signalers over time, with 30 replications (in grey). (Mean counts in black.) Bottom: Intermediate punishment parameter settings ([0.5, 0.5, 0.5, 0.5]): Mean resources of caste 0 2 signalers over time, with 30 replications (in grey). (Mean counts in black.)

We now turn to the signals used and the resources accumulated by agents who use these signals. Fig 10 shows the rate of honest signaling for the low caste agents in the medium regimen. Notice that, while honest signaling increases, there is, as we would expect, a great deal of variance across runs. Fig 10 also shows the resource accumulated by honest low caste signalers. Notice the interesting damping behavior which is recapitulated in the bottom halves of Figs 11 and 12.

**Fig 13:**
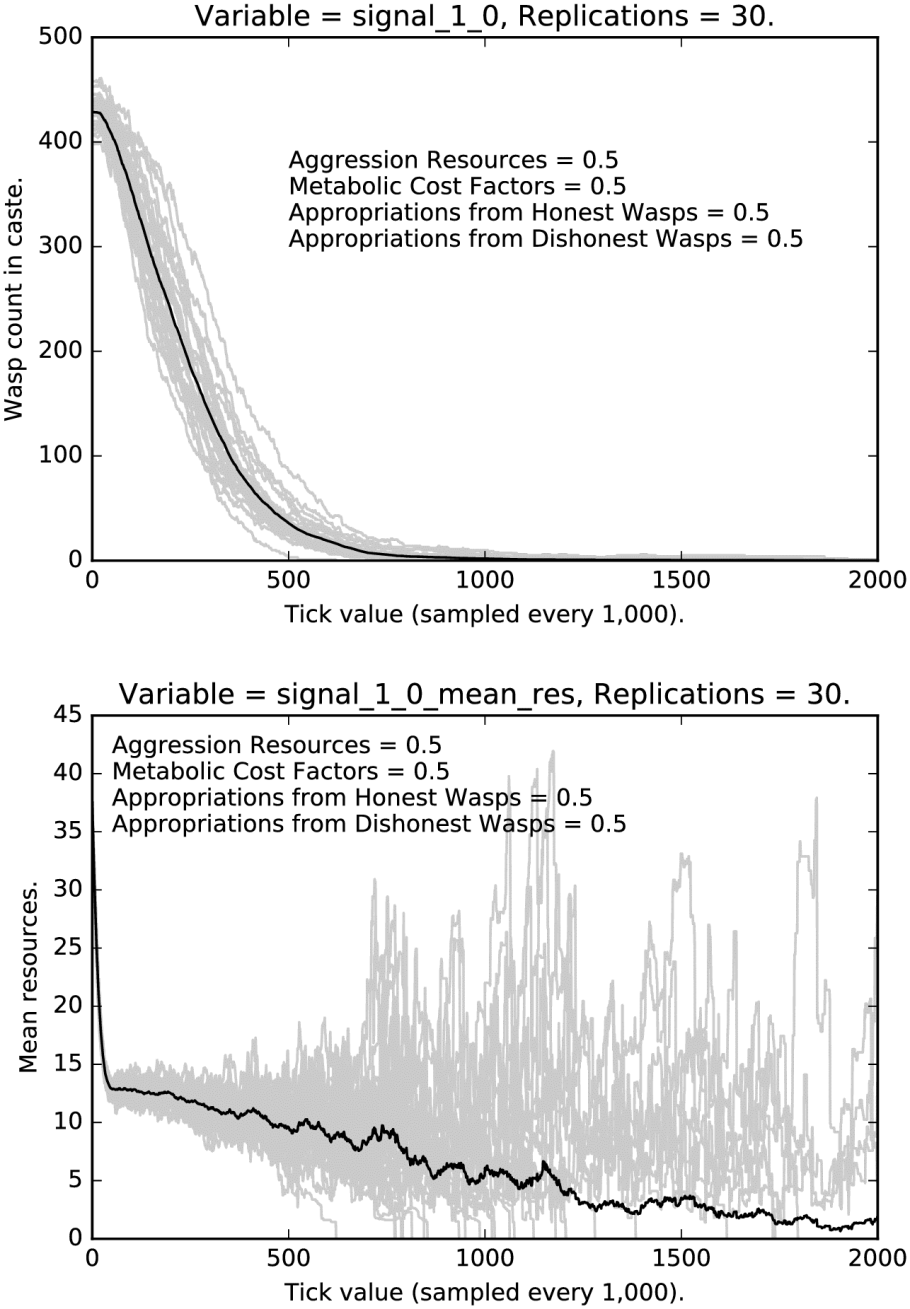
Top: Intermediate punishment parameter settings ([0.5, 0.5, 0.5, 0.5]): Counts of caste 1 0 signalers over time, with 30 replications (in grey). (Mean counts in black.) Bottom: Intermediate punishment parameter settings ([0.5, 0.5, 0.5, 0.5]): Mean resources of caste 1 0 signalers over time, with 30 replications (in grey). (Mean counts in black.)

**Fig 14:**
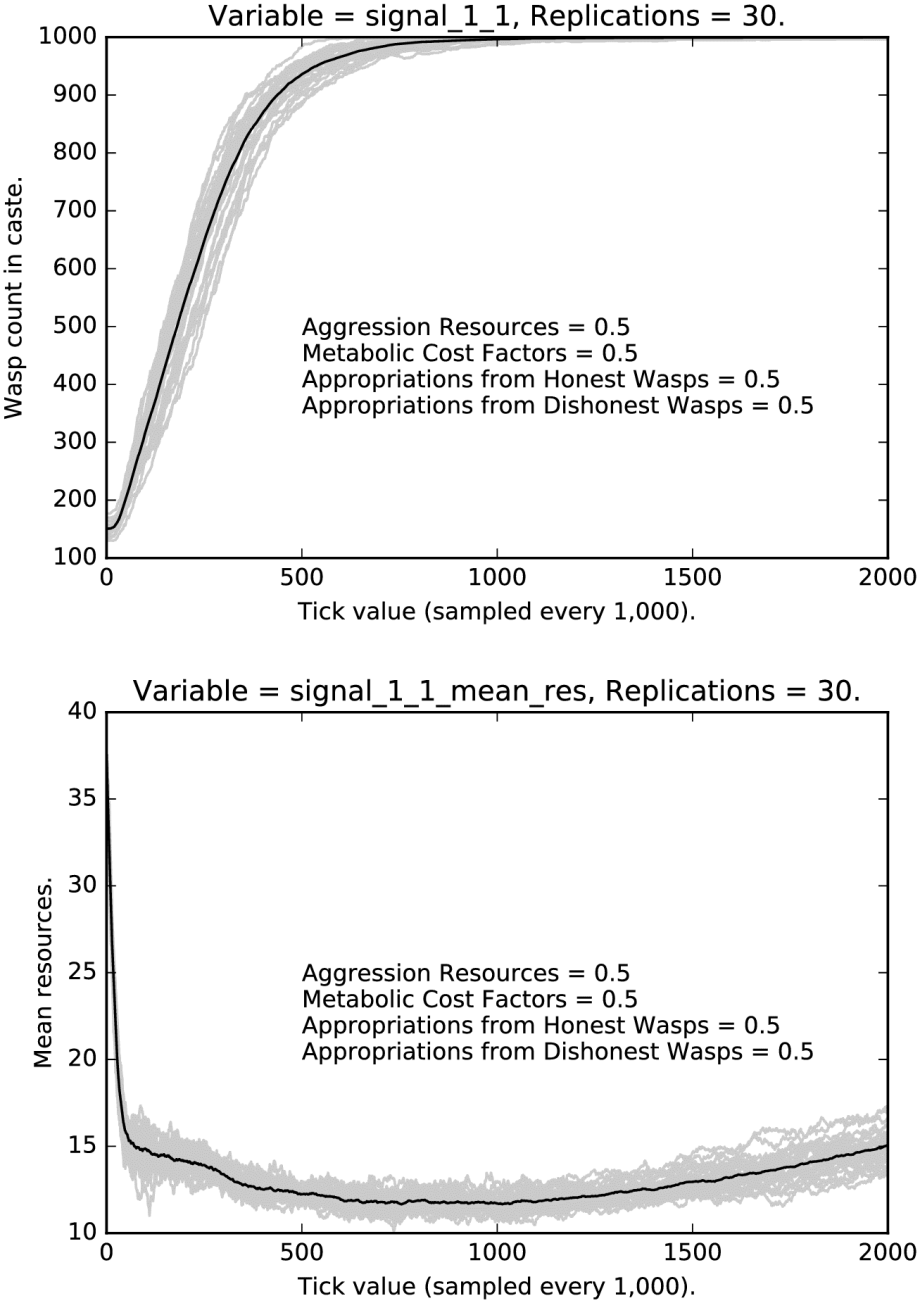
Top: Intermediate punishment parameter settings ([0.5, 0.5, 0.5, 0.5]): Counts of caste 1 1 signalers over time, with 30 replications (in grey). (Mean counts in black.) Bottom: Intermediate punishment parameter settings ([0.5, 0.5, 0.5, 0.5]): Mean resources of caste 1 1 signalers over time, with 30 replications (in grey). (Mean counts in black.)

Turning to the middle caste, Fig 13 shows the rate and mean resources of middle caste agents deceptively signaling that they are low caste agents, while Fig 14 shows the rate and mean resources of honest signalers. Notice that honest signaling quickly dominates the population, and it does so with extremely low variance. Notice that the top panel in Fig 14 mirrors the curve shown in Fig 2, although the curve is gentler, indicating that selection is, unsurprisingly, not as strong in this case. Turning to the mean resources of middle caste honest signalers, note that the mean resources initial drop off quite quickly, then continue to decrease at a gentler rate, then slowly rebounds; the variance is quite small.

**Fig 15:**
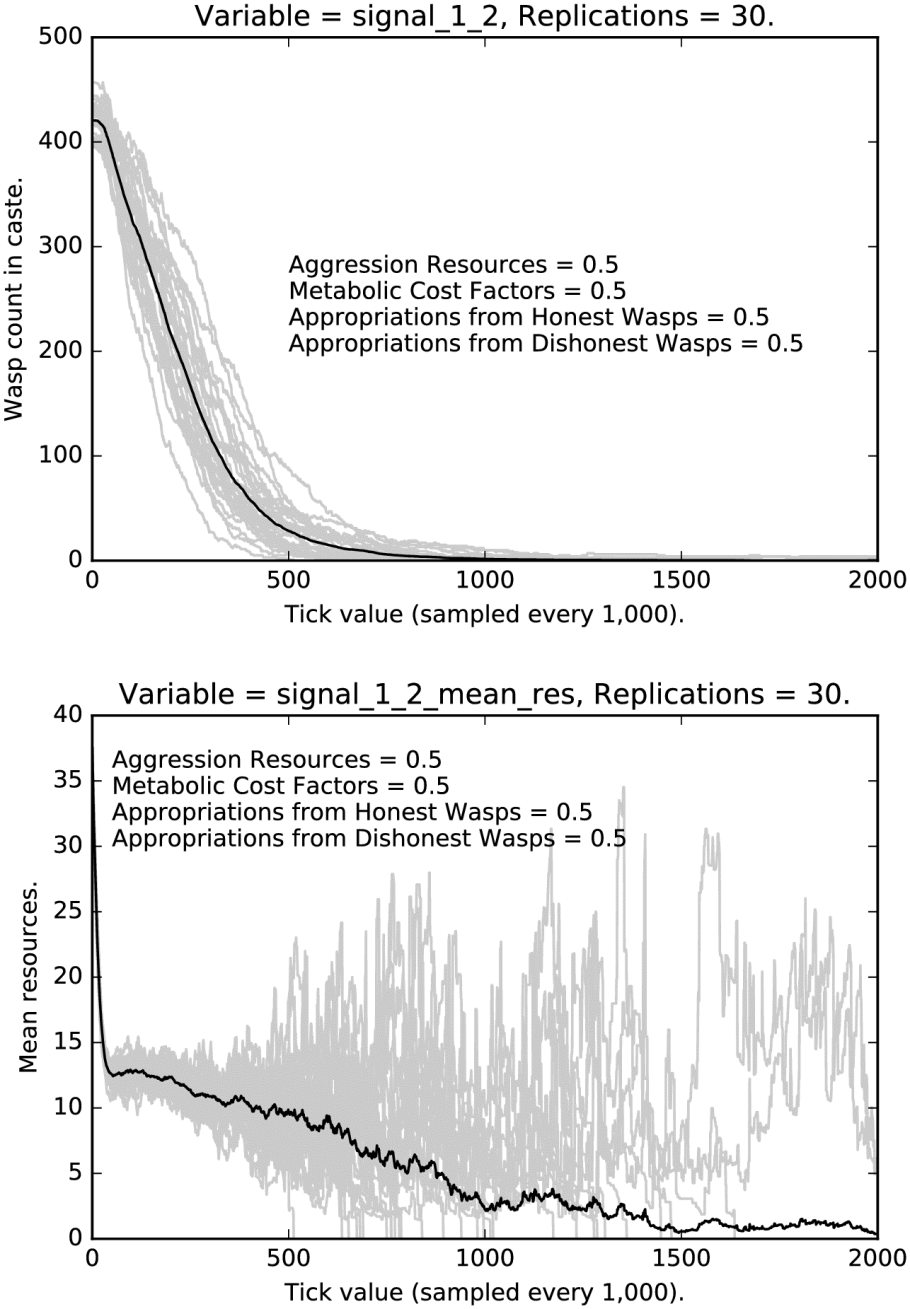
Top: Intermediate punishment parameter settings ([0.5, 0.5, 0.5, 0.5]): Counts of caste 1 2 signalers over time, with 30 replications (in grey). (Mean counts in black.) Bottom: Intermediate punishment parameter settings ([0.5, 0.5, 0.5, 0.5]): Mean resources of caste 1 2 signalers over time, with 30 replications (in grey). (Mean counts in black.)

Fig 15 shows the rate and mean resources of middle caste agents deceptively signaling that they are high caste. Note that in both types of deception (lower, Fig 13; upper, Fig 15), deceptive signaling ultimately vanishes, which means that the resources associated with deceptive signaling also eventually go to zero. Note, however, the high variance in the resources associated with deceptive signaling, which indicates some degree of path dependence. The patterns for both signal 0 deception and signal 2 deception are similar to each other. Recall that the patterns for low caste deceptive signaling, while quite different from the middle caste deceptive signalers, were again similar to each other, which brings out how the pattern of signaling is contingent upon the social structure.

**Fig 16:**
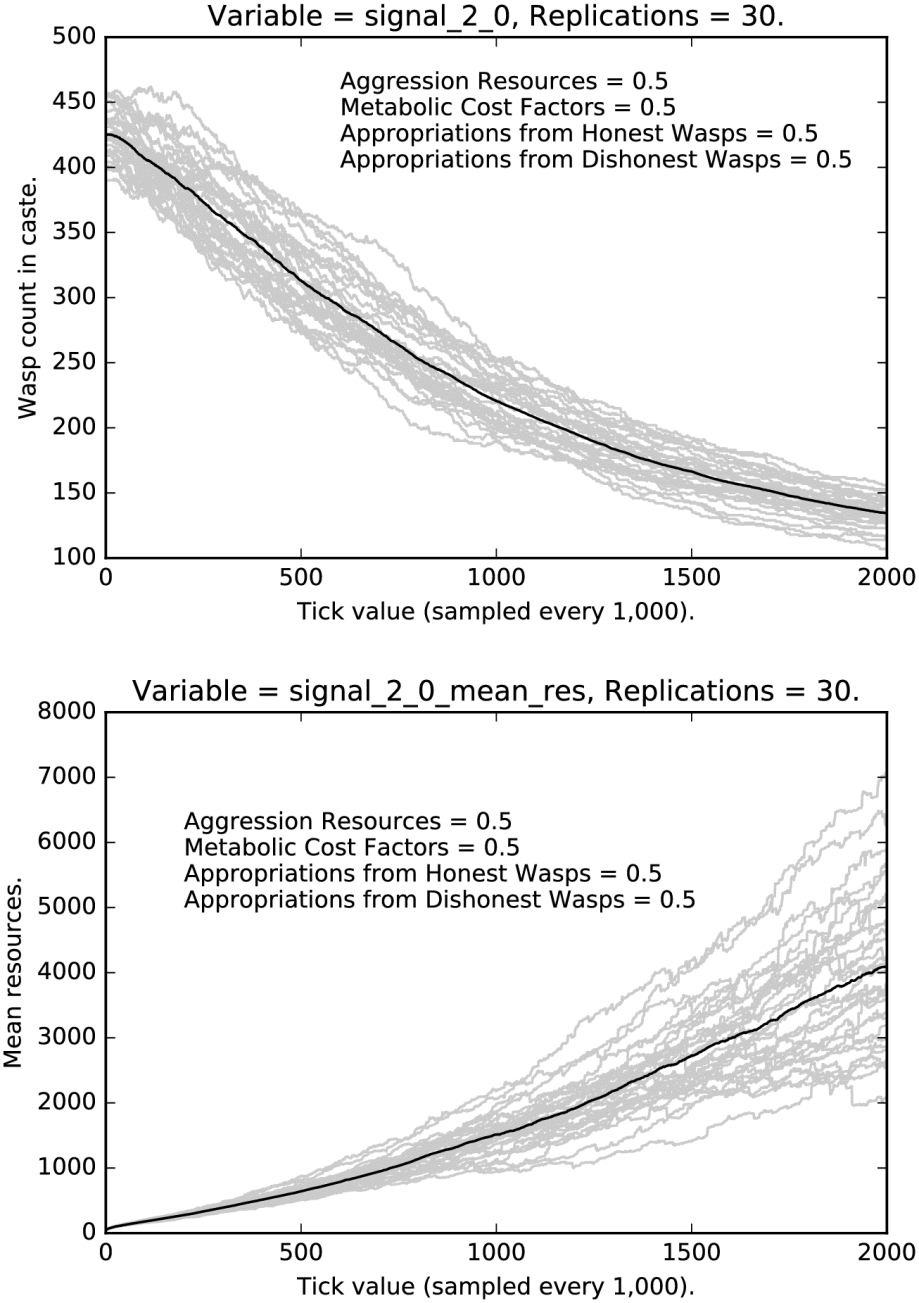
Top: Intermediate punishment parameter settings ([0.5, 0.5, 0.5, 0.5]): Counts of caste 2 0 signalers over time, with 30 replications (in grey). (Mean counts in black.) Bottom: Intermediate punishment parameter settings ([0.5, 0.5, 0.5, 0.5]): Mean resources of caste 2 0 signalers over time, with 30 replications (in grey). (Mean counts in black.)

**Fig 17:**
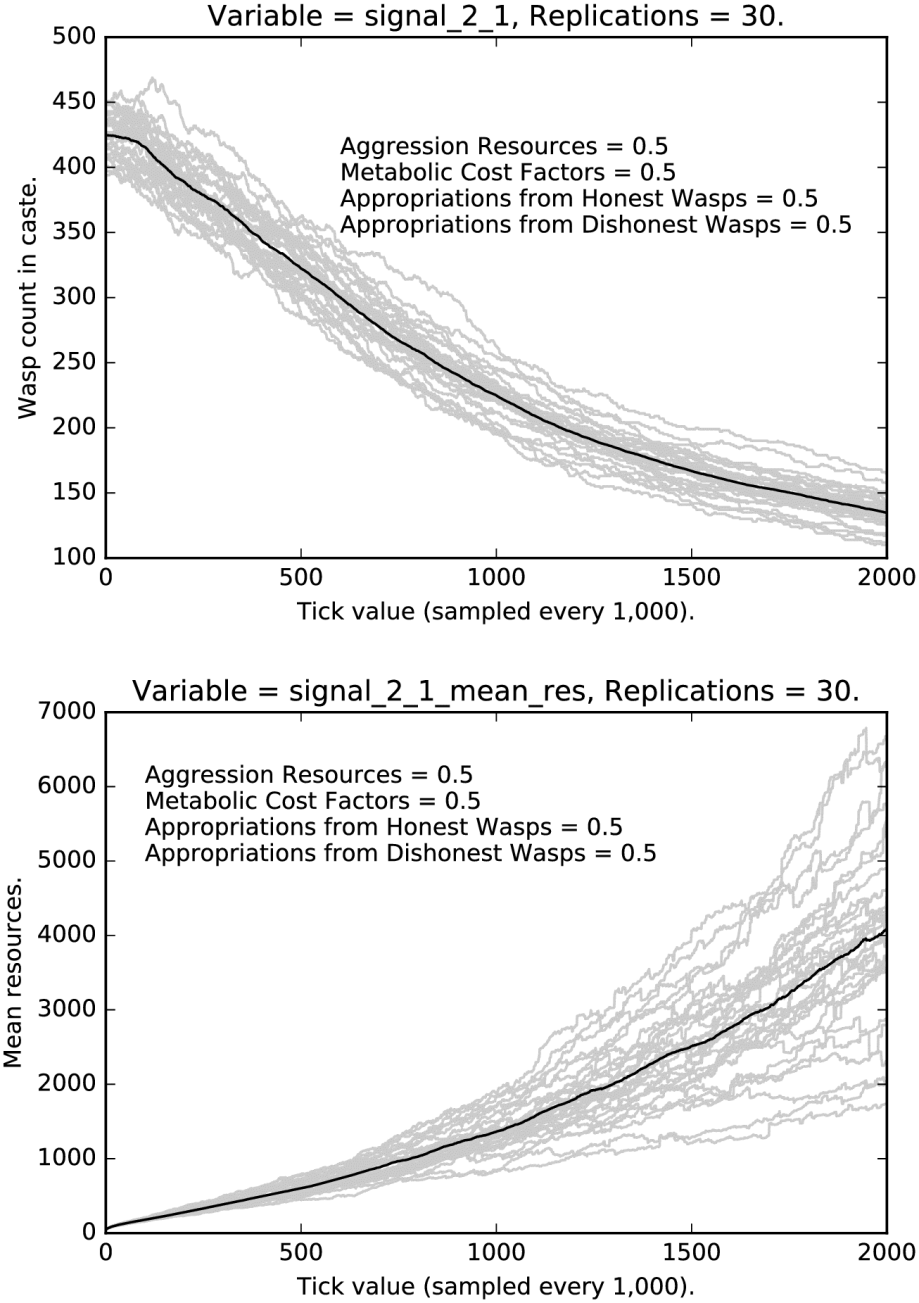
Top: Intermediate punishment parameter settings ([0.5, 0.5, 0.5, 0.5]): Counts of caste 2 1 signalers over time, with 30 replications (in grey). (Mean counts in black.) Bottom: Intermediate punishment parameter settings ([0.5, 0.5, 0.5, 0.5]): Mean resources of caste 2 1 signalers over time, with 30 replications (in grey). (Mean counts in black.)

**Fig 18:**
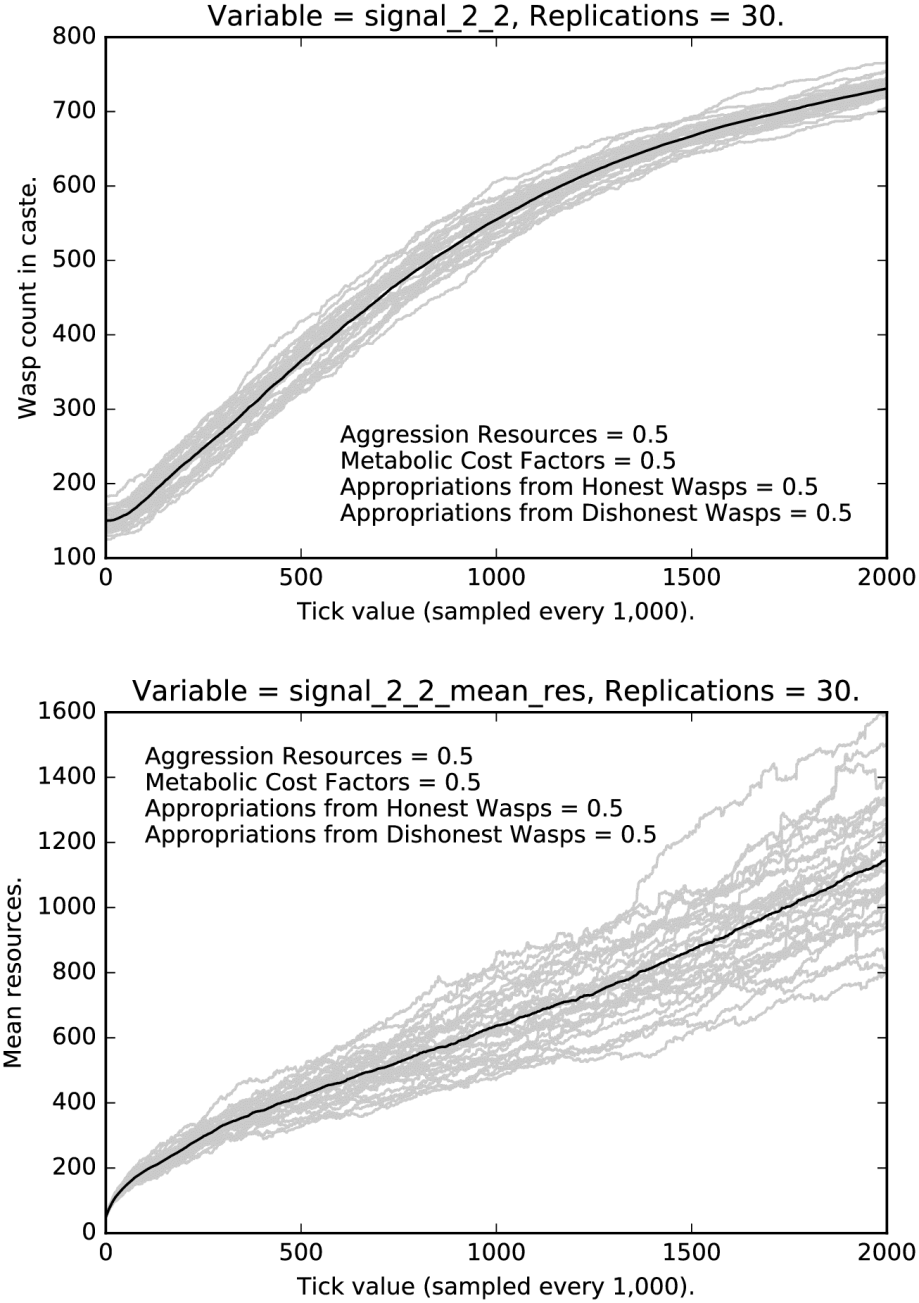
Top: Intermediate punishment parameter settings ([0.5, 0.5, 0.5, 0.5]): Counts of caste 2 2 signalers over time, with 30 replications (in grey). (Mean counts in black.) Bottom: Intermediate punishment parameter settings ([0.5, 0.5, 0.5, 0.5]): Mean resources of caste 2 2 signalers over time, with 30 replications (in grey). (Mean counts in black.)

Finally, let us turn to the highest caste of agents. Figs 16 and 17 show the rates and resources for deceptive signaling among the high caste agents. In these cases, we see a steady decline (but certainly not extinction) of dishonest signaling with a moderate amount of variance. Note that the rate of deception in this case is significantly higher than the rate of deception among low caste agents and the variance is somewhat lower than the low caste case. Notice, also, that the resources of the deceptive high caste signalers go up significantly over time. Compare this last case with the resources of the honest high caste signalers, shown in Fig 18; the mean resources of deceptive signalers are three to four times as high as the mean resources of honest signalers.

## Discussion

We have presented here a stylized model of a social system, loosely based on work on paper wasps [1, 2]; unlike wasps, our agents live in a three-tiered caste system which allows agents in the middle to either bluff—pretend that they are high caste and, thus, scare away potential opponents—or seduce—pretend that they are lower caste, which should allow them to attract and defeat weaker opponents. In this system, we have put aside memory, reputation and any basis for kin selection in favor of an untempered look at the effects of payoffs and punishments in isolation. The original question was whether punishment of dishonest signalers was adequate to guarantee the evolution of honest signaling. We have seen that completely purging deception requires an extremely stringent punishment schedule and a good deal of time. Under more moderate punishment, deceptive signaling is not driven out of the population. This suggests, at the very least, that under moderate and light punishment a mixed equilibrium is possible. In other words, there is a niche for deceptive signaling so long as it is not too frequent in the population. Even more interesting, and for us an unanticipated consequence of the model, is that deception is conditioned by social structure.

The conventional view in the field is that truth is the standard by which all else is measured in the study of linguistic meaning. Linguistic semantics bases its analysis of linguistic meaning on truth conditions; it is standard to characterize the meaning of a sentence based on the possible worlds in which the proposition expressed by the sentence is true. The truth-conditional approach to meaning is paradigmatically associated with [23] and received an influential exposition in [24]; [25] is an up-to-date introduction. [26] is a standard introduction to linguistic pragmatics, although it predates the important work of [27] and [28], both of whom have a much braoder perspective on meaning that attends to more than just information transfer. The latter has been particularly influential in recent work on animal communication [29]. Truth conditions give us a mathematically straightforward account of how linguistic signs are connected to the world and are, therefore, taken as a plausible foundation for linguistic meaning. Clearly, the connection between truth and the world is supported in a system that is, for the most part, honest. In the main, linguistic treatments of meaning have concerned themselves with language as a system for smoothly transferring information from one agent to another. As noted in the introduction, there is a continuity between animal communication systems and human communication with regard to honest signaling. This perspective on meaning has been codified in Gricean pragmatics [3], which treats communication as a form of cooperative behavior. In a nutshell, Grice takes normal conversation to be highly cooperative; each participating agent seeks to make his or her contribution to the conversation appropriately informative. This is the intent of his *Cooperative Principle*. The Cooperative Principle is supported by a number of *maxims*. Of particular interest for present purposes is the *Maxim of Quality*:

**Maxim of Quality**
Try to make your contribution one that is true.
1. Do not say what you believe to be false.
2. Do not say that for which you lack adequate evidence.

Grice takes the Maxim of Quality to be a fundamental fact about cooperative behavior in general. To take his example, if two people are cooking together and one asks for sugar, then a genuine, non-spurious contribution would be for the second person to produce sugar and not salt. The latter would, in some sense, violate the Maxim of Quality, in that the response is not usefully connected to the request. In general, the Maxim of Quality is intended to directly connect language to action in the world via truth conditions. As a descriptive principle, however, the Maxim of Quality falls short; it requires that linguistic agents be invariably truthful, but observation shows that language can be used deceptively. Thus, the Maxim of Quality seems to describe an ideal state and not the facts on the ground.

The system we have developed here explicitly models a force—social punishment—that doubtless acts to promote honest signaling as opposed to merely stipulating honesty on the part of signalers. Nevertheless, we have found this force to be an inadequate guarantee of honesty; deception long persists under any reasonable schedule of punishment. As an alternative, we might suppose that speakers are more interested in the strategic effect that their utterances than in the preservation of truth conditions [7, 8]. This approach is, in fact, consistent with early work in Speech Act Theory [30], where language is viewed as an instrument of action that can be used to influence as well as inform. In this setting, speakers are largely concerned with influencing hearers’ behavior using language as a tool. The preservation of truth, here, is orthogonal to the main goal of persuasion and influence. Signalers are free to use deception in order to achieve their goals, and, naturally, receivers are free to ignore signals they suspect of being deceptive. Natural language seems to be a “cheap talk” system [9] so it should be subject to potentially widespread deception. Of course, real wasps (as well as our models of them) are not using language; they are merely signaling, and they cannot be said to have goals or intentions. Our point is that we can interpret behavior as involving signaling, dishonesty, and so on, without the slightest recourse to Gricean pragmatics. Our suggestion is that perhaps much of human discourse can be similarly interpreted as functionally arising to serve needs and interests rather than truth.

Total deception, however, would render the system useless; listeners would have no reason to attend to the signal sent by speakers, who would, as a result, have no reason to speak. In game theoretic terms, the only response to any signal would be the pooling response; in other words the system would be incapable of transmitting information. Exactly this point has been argued by researchers in animal communication [4, 6]. We need not suppose, however, that a signaling system is used in either an entirely honest or a completely deceptive manner. Our model suggests that, depending on the level of punishment, agents can find a mixed equilibrium combining honest signaling with deception. Agents can find a niche where there is enough honest signaling to keep the system credible (or perhaps sufficiently valuable to maintain), but where some extra utility can be eked out through occasional deception. Deception must be considered an almost constant factor in communication. Useful signaling systems need not be wholly honest signaling systems. Agents can usefully signal to each other, while still using deception to gain advantage, so long as the deception does not overwhelm honest signaling. The system will then retain its utility. This in turn suggests that communication is not simply a matter of transmitting information, but rather involves the ability to elicit a desired response from the receiver, as argued by [7] and [8]. All of this suggests that the basic Gricean project, valuable though it is, needs thoroughgoing revision. This is a project we leave for future research.

Another hypothesis suggested by this work is that the apparent equilibrium of (in most cases) completely honest signaling may in fact never be reached. The fact that the process is so lengthy indicates that exogenous events or countervailing factors that need not be very strong could move the long term equilibrium to the kinds of mixtures we see in our simulations.

A number of additional computational and laboratory experiments suggest themselves. First, can the simple agents in our experiment evolve a signaling system from a random state, given that they are sensitive to aggression level and markings? Here the focal agent would punish an agent in the same caste who uses a different signal from the focal agent; further, it would punish an agent in a different caste who uses the same signal it uses. In a variant, agents might do all the above while developing expectations (in the form of Bayesian learning) about the signaling protocol of other castes and punishing violators based on their evolving expectations. In a more complex (and realistic) variant, there would be conditions for an ecology of norms that, itself, would be subject to evolution. Of course, adoption of a norm for honesty might be undermined by free-riding and so another norm might develop, a meta-norm which has a norm as its object; this meta-norm would establish sanctions against disobeying the first norm, making violation of the first norm unprofitable. We might see an ordered adoption of these norms, for either honesty or dishonesty, resulting in a ratchet effect. A norm for punishing dishonest signalers would be one example but there might be other norms that would have similar outcomes. The general pattern is that selection acting on a rich possibility space may effect a sequence of changes that become something of a ratchet, more difficult to undo once done.

We could model the addition of other properties to the agents so that we can observe the effects of these properties on their signaling behavior in isolation. As noted above, our simple agents have no memory for past behavior and, therefore, cannot track reputation, although we know that reputation can have large effects on behavior [31]. A further experiment would endow the agents with memory and the ability to discriminate the identities of other agents. While a perfect memory might make deception a good deal more difficult, limited memory might allow for deception to persist. Finally, we might allow some form of group identity might have consequences for deception within and across castes (see [32] for some experimental evidence on human subjects).

One next step is to take the model to the laboratory so that we can observe the behavior of subjects in the same circumstances as the agents in our model. For example, subjects could be assigned a “role” (caste, signaling protocol and resources) and, given several rounds of anonymous one-shot play with other agents over a network, decide on a new signaling strategy. The information provided to subjects could be manipulated in the various experimental conditions, as in the computational model. We could, for example, include conditions that not only test for memory and reputation, but also empathy [33], and identity (perhaps in the form of “caste” solidarity).

**Fig 19:**
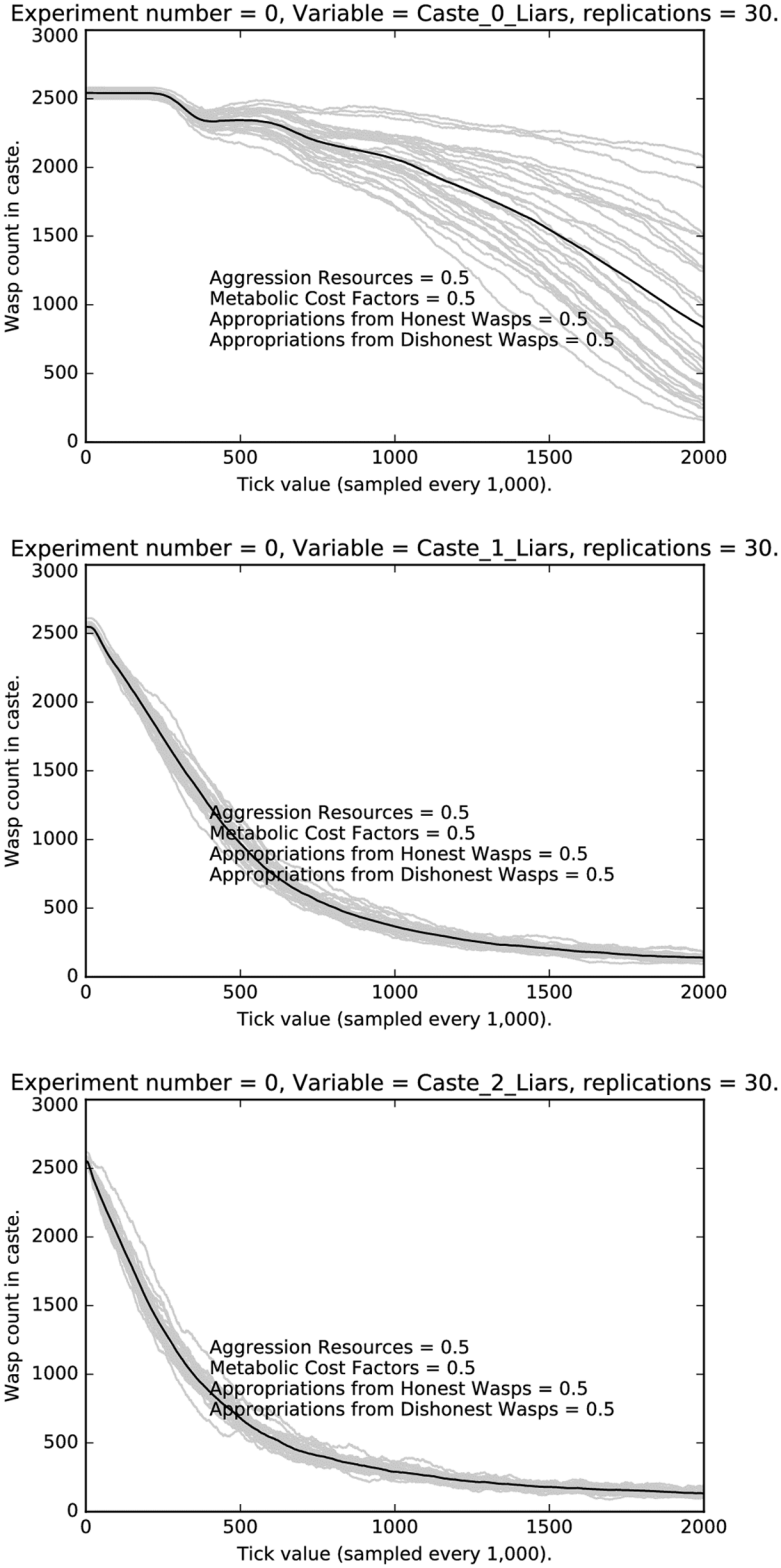
Deception rates in egalitarian societies of caste 0 (Top); caste 1 (Middle); caste 2 (Bottom).

Finally, the item of greatest interest here is the contribution of social structure to deception. We hypothesize that alternative social structures should have an impact on deception rates, even in our simple model. While we can and are developing models with alternative social systems, we hope to test these models with human subjects in a lab setting as well as consider the impact of social structure across cultures. Would a more egalitarian society decrease the rate of deception, for example? As a preliminary step, we have run the simulation on three egalitarian societies, one consisting of only caste 0 agents, one consisting of only caste 1 agents and one consisting of only of caste 2 agents. If social structure is important in conditioning deception rates across castes, then these differences should largely disappear in the egalitarian societies. We show the results in Fig 19. Note that the deception rates for Castes 1 and 2 are essentially identical. The deception rate for Caste 0 agents declines at a lower rate and with more variability due to the reduced level of punishment compared to the other castes, but the effect of social structure has been removed; compare the Caste 0 rates in Fig 19 with the Caste 0 rates with punishment in Fig 7. This suggests that, indeed, social structure interacts strongly with deception.

## Conclusion

To summarize, we have modeled a strategic situation involving deception and punishment in terms of a stratefied population, as is common in evolutionary game theory. The population modifies its behavior via the death of agents and the replication of more successful strategies. Fundamentally, the question we address is whether or not niches may exist for deceptive behavior even in the presence of very strong punishment. What we find is that indeed such niches may exist when different social levels (classes or “castes”) are also present. Further, these niches are filled differentially by different classes. It emerges from our model that the middle caste cannot support deception but the upper and lower castes can and do.

Stepping back and looking into the larger context, the study we describe here belongs to a very broad literature seeking explanations for social phenomena, whether static or dynamic. In general, such social explanations have several kinds of factors that they can marshal. These include, first, the physical environment and even mathematical factors—the real world—which constrain the possibilities for any social order. D’Arcy Wentworth Thompson’s *On Growth and Form* [34] has been influential in introducing these kinds of considerations into biological explanation. Recent work in niche construction, e.g., [35], might be classified here as well. Second, there are biological factors, for example accounts that evoke selection and evolution to explain the phenomena to hand. Culture is a third explanatory factor, one that has received increasing attention of late. Advocates of cultural factors have often sought to contrast them with biological factors, for example as the following passage indicates.

Thus, the three common explanations for our species’ ecological success are (1) generalized intelligence or mental processing power, (2) specialized mental abilities evolved for survival in the hunter-gatherer environments of our evolutionary past, and/or (3) cooperative instincts or social intelligence that permit high levels of cooperation. All of these explanatory efforts are elements in building a more complete understanding of human nature. However, as I’ll show, none of these approach can explain our ecological dominance or our species’ uniqueness without first recognizing the intense reliance we have on a large body of locally adaptive, culturally transmitted information that no single individual, or even group, is smart enough to figure out in a lifetime. To understand both human nature and our ecological dominance, we first need to explore how cultural evolution gives rise to complex repertoires of adaptive practices, beliefs, and motivations. [36]

Social structure, in our view, constitutes a fourth kind of factor in social explanations, one that has received modest, but very intriguing, attention (see, e.g., [37] for a biologically-oriented study and [38] for a sociological perspective). It is social structure that has been the focus of our study, which explores the role of social structure in maintaining dishonesty in the face of punishment for lying; we intend this study to be a contribution to the broader literature on the evolution of social structure [39–41]. Understanding the evolutionary construction of social structure in conjunction with punishment has been an active area of research [42], although the investigation remains very much open.

**Fig 20:**
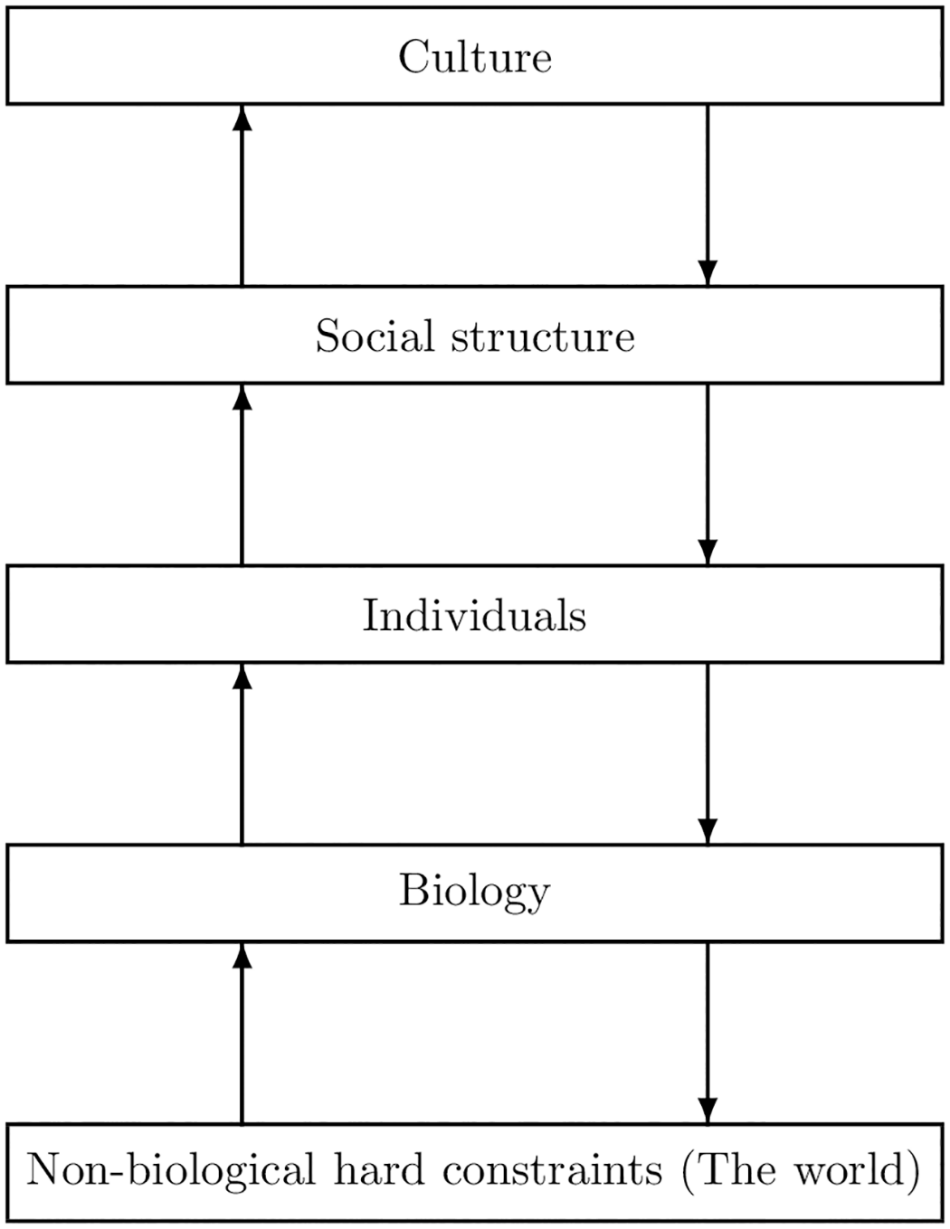
Alternative schematic of factor interactions.

Fig 20 may be taken as a schematic representation of these explanatory factors. The thought is that the social order emerges from the behavior of individuals, but this behavior may be conditioned in complex ways upon culture, social structure, biology, and the environment.

One way to think of the importance and role of social structure is that there is a real connection between social structure and biology; that is, some form of dual inheritance theory accounts for the mixture of caste and rates of deception that we see in these simulations. For example, powerful high caste agents arrive at an equilibrium state where the rate of deception is relatively elevated; low caste agents arrive at an equilibrium that also has an elevated rate of deception, though not as high as the most powerful agents; middle caste agents, squeezed from above and below, do best when their signaling is honest. These states are the result of ameliorating their gains and costs, relative to the system of punishment and social structure that they find themselves in.

These and related matters, including systematically discerning the interactions and influences of the several factors shown in Fig 20 and developing a niche-oriented account of signaling systems (where the niche is defined in terms of the interests and needs of the participants). This must await future research. It is remarkable how a carefully considered model of punishment among agents has opened up such far ranging issues.

We would hypothesize that real societies would be influenced by the simple forces of punishment and social stratification, which we model in this paper. Of course, other factors may intervene, so ultimately the question can only be resolved by developing more sophisticated models (including, for example, models including reputation and individual learning) and by conducting experiments with living subjects.
